# Fundamental features of social environments determine rate of social affiliation

**DOI:** 10.1073/pnas.2506243122

**Published:** 2025-10-14

**Authors:** Sankalp Garud, Miruna Rascu, Sorcha Hamilton, Ingrid Yu, Matthew F. S. Rushworth, Miriam C. Klein-Flügge

**Affiliations:** ^a^Oxford Centre for Integrative Neuroimaging, Department of Experimental Psychology, University of Oxford, Oxford OX1 3SR, United Kingdom; ^b^Department of Psychology, University of Bath, Bath BA2 7AY, United Kingdom; ^c^Department of Psychiatry, Warneford Hospital, University of Oxford, Oxford OX3 7JX, United Kingdom

**Keywords:** social neuroscience, mental health, foraging

## Abstract

Humans start new friendships and social connections throughout their lives and such relationships foster mental and physical well-being. While friendship initiation may depend on alignment of subtle and complex personal variables, here we investigated whether it also depends on basic features of social environments. In a preregistered online study (n = 783) using a novel social-affiliation seeking paradigm, we found people were more likely to send friend requests as the density of friendship opportunities decreased and frequency of success increased. Further, we found task-related measures, like overall friend requests, were correlated with mental health dimensions like social thriving and anhedonia. Next, in an ultra-high-field fMRI study (n = 24), we found that both fundamental features of social environments--opportunity density and frequency of success--affected neural activity across a network of regions linked to foraging including dorsal raphe nucleus, substantia nigra, and anterior insula. Thus, humans consider the background statistics of an environment while making social decisions and these decisions are linked to activity in cortico-subcortical circuits mediating the influence of environmental statistics on other aspects of behavior. Moreover, individual differences in how environmental features influence social behavior are associated with variation in mental health dimensions, offering key insights into interindividual variability in social functioning.

## Significance

Social connection is important for our well-being, but little is known about the environmental factors and brain mechanisms that enable its initiation. In an experimental task, we show that two fundamental features of social environments, how friendly people are in that environment and how frequent opportunities for social connection arise, shape decisions to initiate friendships with others. In addition, people’s sensitivity to the social environment related to their mental health dimensions such as their social thriving. Using ultra-high field MRI, we show that a cortico-subcortical neural circuit responsible for tracking rewards during foraging also tracked features of social environments. This suggests that initiating social connections is linked to evolutionarily conserved brain mechanisms, underscoring the fundamental nature of social affiliation.

Social connections positively impact human well-being. For instance, increased social contact is a major predictor of happiness, whereas increased loneliness and isolation are linked to feelings of depression and increased mortality risk (1–4). Thus, it is important to understand how social connections start and what neural mechanisms support their formation.

Although from a personal perspective social connections are freighted with meaning, social psychologists have identified a series of factors such as similarity, familiarity, and proximity that facilitate connection between individuals (5). It has also been argued that social connection might be a basic human need and even that, at the level of the brain’s motivation circuits, the impact of social deprivation resembles that of food deprivation (6, 7). If this is the case, then we might expect that the initiation of social connection is influenced by other factors that equally reflect little about the specific characteristics of the individuals involved. For example, if social connection is as much of a “fundamental need” as is food, then insights into how we initiate social contact might be gleaned from how humans and animals forage for food.

Classical and modern studies of foraging have suggested that beyond the value of a food opportunity in itself, animals take into account background statistics of opportunities in an environment (like average rate of reward) when deciding whether to engage with any opportunity (8–10). In an analogous manner, here we hypothesized that decisions about whether to initiate social engagement with an individual might depend on similar fundamental features of the background statistics of social environments. That this might be the case is also suggested by the way in which other human decision-making problems have also been shown to share features with common foraging problems (11–14). Concretely, drawing on investigations into foraging (8–10, 15), we expected two features of social environments would influence people’s decisions to initiate friendships: i) the average friendliness of an environment, that is, the average success of one’s attempts to create friendships in a given environment, and ii) the opportunity density, that is, the number of opportunities one gets to create friendships in a given environment. We found that this was indeed the case.

Given the link between social isolation and mental well-being (1–3, 16), we also carried out a second line of investigation, examining the relationship between the impact of the statistics of social environments on people’s tendencies to initiate social contact and their mental health profile. To assess state and trait mental health, we asked people to complete standardized questionnaires that measure symptoms for conditions ranging from depression to anxiety and loneliness. These items were selected to span a variety of social and nonsocial trait measures—some we expected to be relevant for social affiliation seeking (e.g., loneliness, anhedonia), and others included as potential control measures (e.g., obsession, compulsion). We used factor analysis, a data driven dimensionality reduction approach, to identify latent mental health dimensions (17), We then examined how these transdiagnostic mental health dimensions were related to the impact of social environments on seeking social affiliation. All behavioral hypotheses were preregistered based on a smaller discovery sample (n = 218) and then replicated in a larger confirmatory sample (n = 767).

A third line of investigation considered the neural mechanisms through which affiliation decisions were influenced by statistics of the social environment. We focused on a cortico-subcortical circuit implicated in the tracking of the background statistics of reward environments during foraging (9, 10, 18–26) to test whether they played an analogous role in social affiliation seeking. We investigated activity across a distributed cortico-subcortical circuit comprising the dorsal raphe nucleus, substantia nigra, habenula, dorsomedial prefrontal cortex, and anterior insula, and we also examined relationships between social affiliation seeking and individual mental health dimensions.

## Results

### Environmental Friendliness and Density Influence Social Affiliation Choices.

**Fig. 1.**
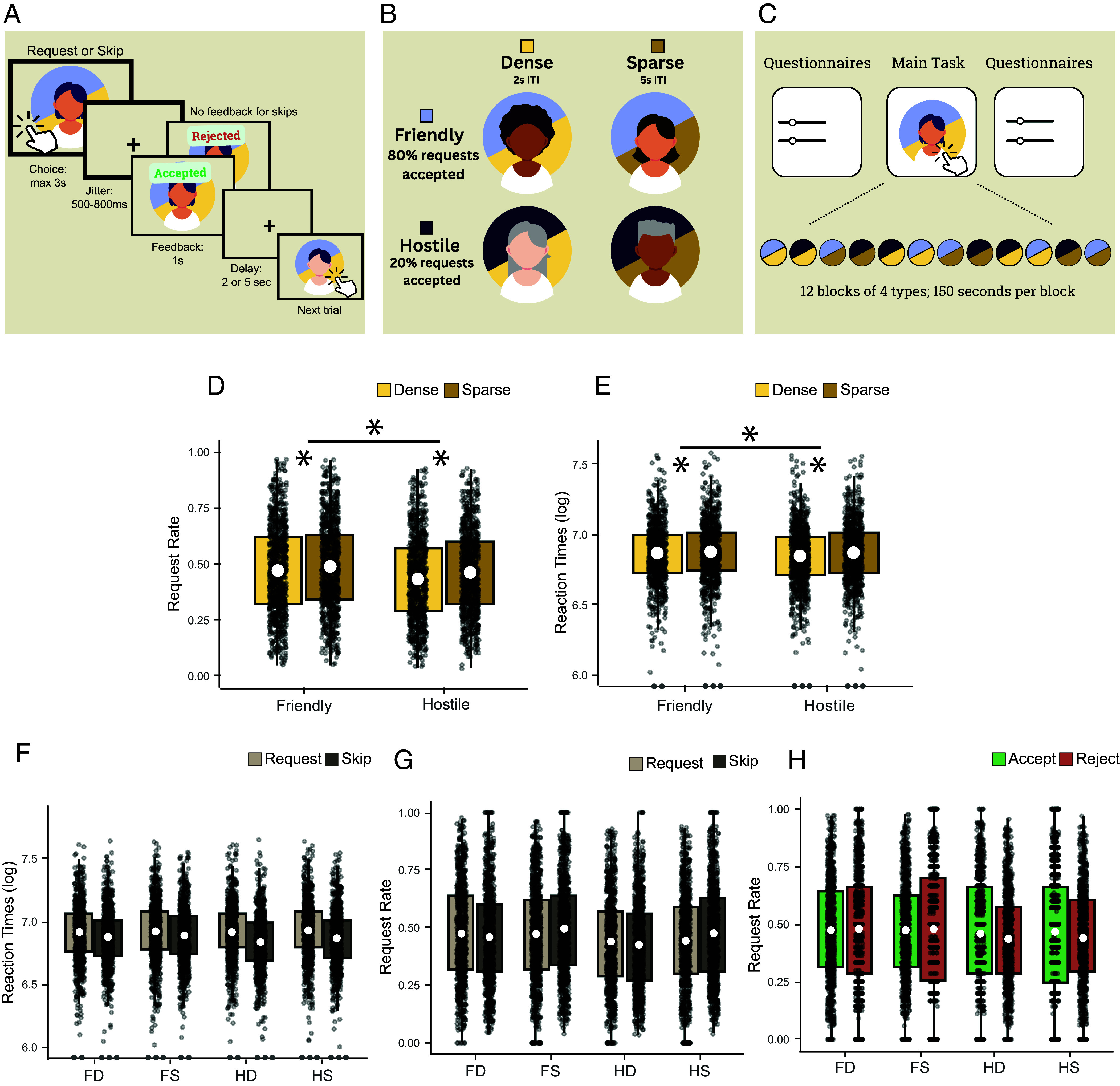
Task structure and behavioral effects in the Friend Request Task. (*A*) schematic representation and timings of a trial. (*B*) Two experimental manipulations, friendliness and density, were used. In a friendly block, 80% of friend requests sent are accepted, while in a hostile block 20% of requests are accepted. In a dense block, the intertrial interval is a shorter 2 s compared to the 5 s interval in a sparse block. (*C*) Overview of the experimental structure in any given run. (*D*) Boxplots indicate the effects of friendliness and density on request rates (the white circle indicates mean; box boundaries indicate interquartile range [IQR], encompassing the middle 50% of the data; whiskers extend to furthest data points within 1.5 × IQR from the box boundaries). The *x*-axis indicates the two levels of friendliness, and *y*-axis indicates the request rate (average number of requests sent) for an individual in that block type. Color indicates the two levels of density. This shows higher request rates in sparser and friendlier environments. (*E*) The effects of friendliness and density on RTs. The Y-axis indicates average log-transformed RTs in the respective block type. RTs are slower in friendly and sparse blocks. Dots shown at the *y*-axis limit denote outliers. (*F*) RTs split into request and skip trials. Color indicates action (request/skip) (*G*) The effect of previous trial action on choice in the next trial. Color indicates action (request/skip). (*H*) The effect of previous trial feedback on choice in the subsequent trial in different environment types. Color indicates feedback received (accept/reject)

To investigate whether social affiliation choices are influenced by fundamental features of the environment, we designed a social affiliation seeking task (“Friend Request Task”). This task, and our hypotheses, were preregistered based on an exploratory sample of n = 218 online participants. To confirm our results in a replication, results from which are presented here, we then asked n = 783 online participants to play the same task again, which involved making repeated choices about whether to send a friend request to faces displayed on the screen (Fig. 1). The cover story asked participants to imagine moving to a new city and making new friends. They were told they would be helped to create connections by being taken to different clubs. Inside each club, participants were shown a series of faces (each face was shown only once) and given the opportunity to send or skip sending a friend request by pressing one button or another. If participants sent a friend request, the next screen would show them whether their request was accepted or rejected (Fig. 1*A*). There was no monetary or temporal consequence associated with either action (skipping or requesting) or outcome (accept or reject).

Importantly, to investigate whether fundamental features would influence participants' affiliation choices, different clubs varied in two respects which was something the participants were explicitly informed about: first, the friendliness of the people in the club and second, the number of people that appeared in the club. As a result, four combinations of clubs were possible: friendly-densely (FD) populated, friendly-sparsely (FS) populated, hostile-densely (HD) populated, and hostile-sparsely (HS) populated (Fig. 1*B*). Participants spent 2.5 min, which we refer to as a block, in one club before moving on to another. In a friendly club, 80% of all friend requests that a participant sent were accepted whereas in a less friendly (more hostile) club, only 20% of all requests were accepted. In a densely populated club, the time between two consecutive encounters with club members was briefer; the faces appeared every 2 s with a jitter between 400 and 700 ms. In a sparse club, the time between consecutive faces was 5 s with an equivalent jitter. The background color associated with each club (blue or green counterbalanced over participants) indicated the friendliness level of the club and the background pattern (smaller or larger circles counterbalanced over participants) indicated the density of club encounters. Participants completed twelve blocks of the task (three per block type) in randomized order.

Our first key analysis focused on whether the two basic features of the social environment manipulated in our task, friendliness and density, affected people’s tendency to send friend requests. A 2 × 2 ANOVA (two levels of friendliness and two levels of density) revealed a significant effect of friendliness on request rates (df = 782, F = 95.7, *P* = 2.17e-21, $\eta_{p}^{2}$ = 0.109; Fig. 1*D*). People were more likely to send friend requests when they were in a friendly environment as opposed to a hostile environment. Similarly, density also had a significant effect on request rates (df = 782, F = 123.5, *P* = 9.77e-27, $\eta_{p}^{2}$ = 0.136). As such, people were more likely to send friend requests when they were in a sparse environment compared to a dense environment. Finally, the interaction between friendliness and density was significant (df = 782, F = 6.7, *P* = 0.01, $\eta_{p}^{2}$ = 0.009). People were more likely to send friend requests in sparse environments, but particularly so when in hostile blocks. These effects were clear in all trials, but also robust to excluding the first ten trials of each block to allow participants to experience the ITI distribution associated with the two levels of density and the acceptance rate associated with the two levels of friendliness (friendliness: df = 782, F = 59.285, *P* = 4.12e-14, $\eta_{p}^{2}$ = 0.07; density: df = 782, *P* = 8.05e-13, $\eta_{p}^{2}$ = 0.063; interaction: df=782, F = 2.340, *P* = 1.26e-01, $\eta_{p}^{2}=0.003$). Preregistered mixed models showed the same results (*SI Appendix*).

### Environmental Friendliness and Density Influence Social Affiliation Reaction Times.

Having identified influences of the social context on affiliation choices, our second key analysis asked whether other aspects of behavior such as RTs, which may reflect aspects of participant’s engagement, confidence, or experienced social pressure in their choice, might similarly reflect features of the social environment. An analogous 2 × 2 ANOVA with factors friendliness and density identified a significant effect of friendliness on RTs (df = 782, F = 38.7, *P* = 8.08e-10, $\eta_{p}^{2}=$ 0.047; Fig. 1*E*); people were slower to respond in friendly compared to hostile environments. There was also a significant effect of density (df = 782, F = 40.22, *P* = 3.84e-10, $\eta_{p}^{2}$ = 0.049); people were slower to respond in sparse compared to dense environments. There was an interaction between friendliness and density (df = 782, F = 13.57, *P* = 2.46e-04, $\eta_{p}^{2}$ = 0.017). People were slower in sparse compared to dense environments, and this effect was especially pronounced in hostile blocks.

### Previous Trial Choice and Feedback Influence Social Affiliation Choices At the Next Opportunity.

It is possible that a previously successful or unsuccessful friendship attempt influences our choice the next time an opportunity arises. For example, a friendship rejection could make us even more cautious, or it could increase the pressure to make it happen next time. Similarly, it is possible that the previous choice has an influence on the next choice we take, for example because of slow fluctuations in whether we generally “feel like” sending requests at the moment. These are the two key questions we addressed in our next two analyses, looking at both RTs and choices at the next opportunity. First, we examined whether the action (request or skip) participants had chosen on the previous trial affected participant behavior on the next trial and how this differed between social environments. Participants’ action on the current trial indeed affected RTs on the next trial (2 × 2 × 2 ANOVA: main effect of action: df = 782, F = 103.2, *P* = 7.32e-23, $\eta_{p}^{2}$ = 0.12; Fig. 1*F*). People were faster if the response was a skip as opposed to a request. There was also an interaction between the response type and friendliness (df = 782, F = 85.4, *P* = 2.31e-19, $\eta_{p}^{2}$ = 0.098). People were faster to skip than to request, but this effect was stronger in hostile blocks compared to friendly blocks. Similarly, there was an interaction between response type and density (df = 782, F = 20, *P* = 8.92e-06, $\eta_{p}^{2}$ = 0.025). People were faster to skip than to request, but this effect was stronger in dense compared to sparse blocks. Thus, RTs also reflected key features of the social environments manipulated in our task.

The previous action (request or skip) also affected how likely people were to send friendship requests on the next trial. An analogous 2 × 2 × 2 ANOVA on request rates (friendliness x density x previous choice) showed a significant interaction between previous choice and density (df = 782, F = 87.4, *P* = 9.21e-20, $\eta_{p}^{2}$ = 0.10; Fig. 1*H*). People were more likely to send a request following a previous request in dense environments, but they were more likely to send a request following a skip in sparse environments. The equivalent test for friendliness interacting with previous action was not performed as the result was not significant in our exploratory analysis, and therefore not preregistered.

Finally, we examined whether social feedback received in one trial differentially affected how likely people were to send friendship requests in the following trial in the different social contexts. We extended the 2 × 2 ANOVA with friendliness and density to include a third factor of previous trial feedback (“accept” vs “reject”). Indeed, there was a main effect of previous trial outcome showing that the outcome received on a previous trial also affected participant behavior in the next trial (2 × 2 × 2 ANOVA with factors friendliness, density, and previous trial feedback: previous outcome: df = 755, F = 5.3, *P* = 2.20e-2, $\eta_{p}^{2}$ = 7.00e-3; Fig. 1*G*). People were more likely to send a request after their previous request was accepted compared to when it was rejected. The interaction between friendliness and previous trial feedback was also significant (df = 755, F = 7, *P* = 8.00e-3, $\eta_{p}^{2}$ = 9.00e-3); people were more likely to send a request following an acceptance, but this was particularly the case in hostile blocks. The equivalent test for feedback interacting with density was not performed because the result was not significant in our exploratory analyses performed in the preregistration cohort, and therefore not preregistered.

### Transdiagnostic Mental Health Dimensions Are Associated with Social Connection Seeking on the Friend Request Task.

Having established that the social environment had an effect on social affiliation choices, in our second line of investigation, we asked whether these influences may be related to mental health dimensions that reflect aspects of social well-being. In addition to the task, participants (n = 783 as above) also completed a series of standardized questionnaires (Methods). Questionnaires were selected to span a variety of social and nonsocial trait measures, including ones we expected to be relevant for social affiliation seeking (e.g., loneliness, anhedonia), and others that we did not expect to relate to task behavior (e.g., obsession, compulsion). Again, all hypotheses were preregistered and the complete set of tests are reported in the *SI Appendix*.

**Fig. 2.**
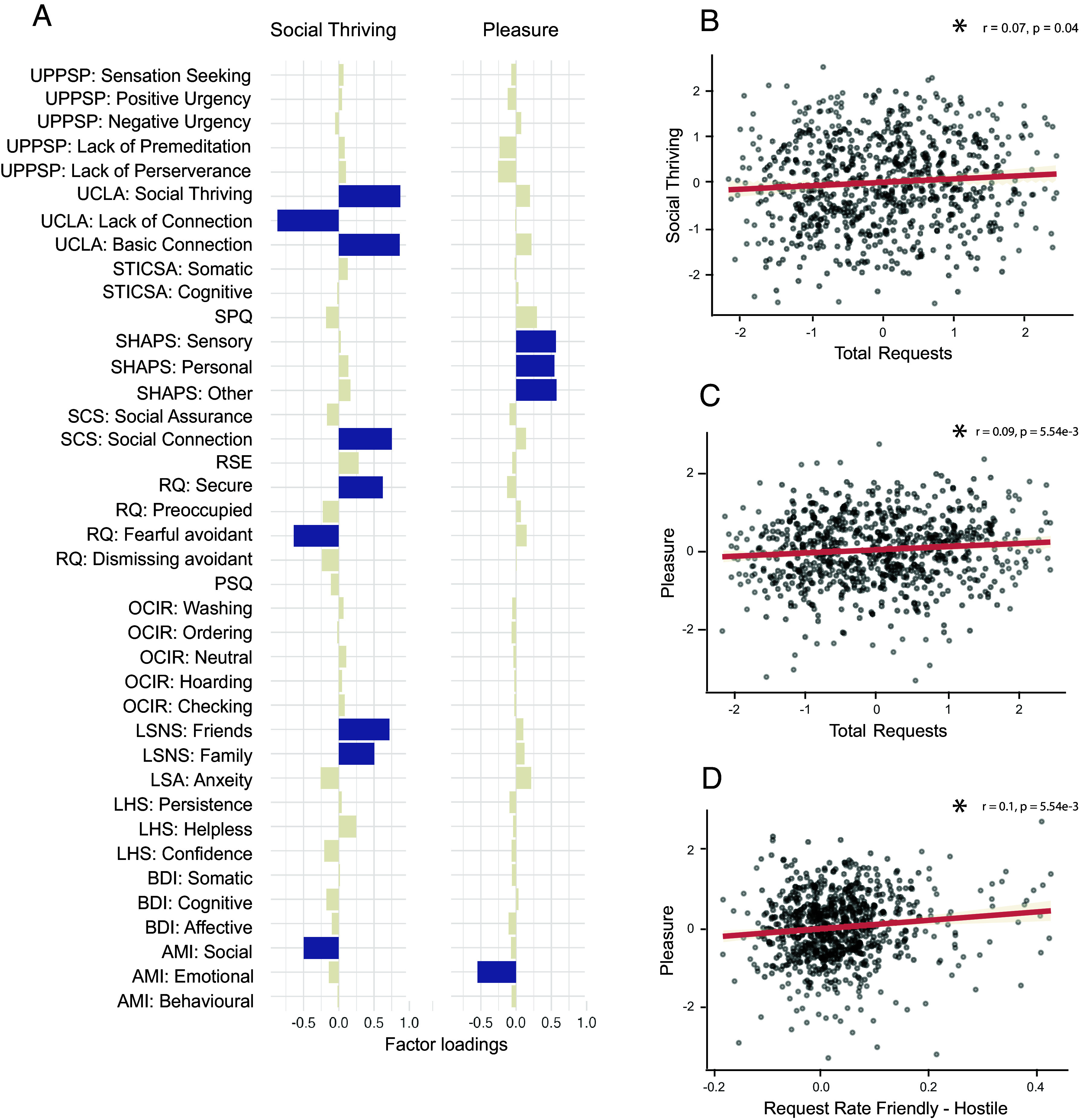
Mental health dimensions loadings and relationship with social affiliation choices in the Friend Request Task. (*A*) Factor structure and loadings of the Social Thriving and Pleasure (reduced Anhedonia) mental health dimension. (*B*–*D*) Scatter plots show the key relationships identified between Friend Request Task measures and transdiagnostic dimensions (these tests were preregistered). For a full table of factor loadings for all seven dimensions, please refer to *SI Appendix*, Fig. S2. UPPSP: Urgency, Premeditation (lack of), Perseverance (lack of), Sensation Seeking, Positive Urgency, Impulsive Behaviour Scale. UCLA: UCLA loneliness scale. STICSA: State-Trait Inventory of Cognitive and Somatic Anxiety. SPQ: Social Pain Questionnaire. SHAPS: Snaith–Hamilton Pleasure Score. SCS: Social Connectedness Scale. RSE: Rosenberg Self-Esteem scale. RQ: Relationship Quotient. PSQ: Pain sensitivity questionnaire. OCIR: Obsessive Compulsive Inventory revised. LSNS: Lubben Social Network Scale. LSA: Liebowitz Social Anxiety Scale. LHS: Learned Helplessness Scale. BDI: Beck Depression Inventory. AMI: Apathy Motivation Index.

Using exploratory factor analysis, we replicated six out of our seven preregistered mental health dimensions. We focused our main analyses on two of the mental health dimensions that were of particular relevance for potential relationships to social affiliation choices (Fig. 2*A*)–Social Thriving, and Pleasure (which also reflects lower Anhedonia, but indicating a positive direction). Social Thriving was positively related to scores on the University of California Los Angeles (UCLA) loneliness scale (version 3) (27) including the Social Thriving subscale and Basic Connection subscale as well as on the Lubben Social Network Scale’s (LSNS) (28) Friends subscale and Family subscale, which assess objective social network size. In addition, the social thriving factor we identified had a negative loading on the UCLA Lack of Connection scale and the Apathy Motivation Index (AMI) (29) Social subscale. Thus, it captured important aspects of people’s social network and connectedness. The Pleasure or reduced anhedonia scale that we identified, had positive loadings for the Snaith–Hamilton Pleasure Score (30) (SHAPS) Sensory, Personal, and Other-related types of pleasure/anhedonia and a negative loading on the AMI (29) Emotion subscale. This dimension captured the overall ability to experience pleasure and to seek out pleasant, emotionally positive experiences.

In the key analysis, we then investigated whether the total requests sent in the Friend Request Task was associated with the identified two mental health dimensions. In line with our preregistration, we found that participants’ social thriving scores were correlated with the total friend requests they submitted in the task (n = 783, r = 0.07, *P* = 0.04; Fig. 2*B*). In other words, people who more frequently requested rather than skipped a friendship opportunity were those associated with higher social thriving scores.

Similarly, the total number of requests made by participants was also related to their pleasure factor score, or in other words, reduced anhedonia symptoms (n = 783, r = 0.09, *P* = 5.54e-3, Fig. 2*C*). Intriguingly, however, the pleasure factor was not just related to the total number of requests made in the Friend Request Task but, in addition, it was related to the impact of a basic feature of social environment – the average friendliness – on social connection seeking (i.e., the request rate difference between friendly and hostile blocks: n = 783, r = 0.1, *P* = 5.07e-3, Fig. 2*D*). In summary, of all the dimensions, the pleasure (or reduced anhedonia) factor, therefore, may be particularly important in capturing how features of the background social environment influence whether people initiate social contact.

Multiple comparisons were not corrected for as the aforementioned tests were preregistered as independent hypotheses. However, after correcting for multiple comparisons, the relationship between the pleasure factor and total request remained significant (p corrected = 7.5e-3), as did the one between pleasure factor and the difference in request rate between friendly and hostile environments (*P* corrected = 2.5e-2). The relationship between social thriving and total requests, however, was no longer significant (*P* corrected = 0.12).

### High-Resolution Neuroimaging of the Neural Circuits Tracking Social Environment Statistics.

**Fig. 3.**
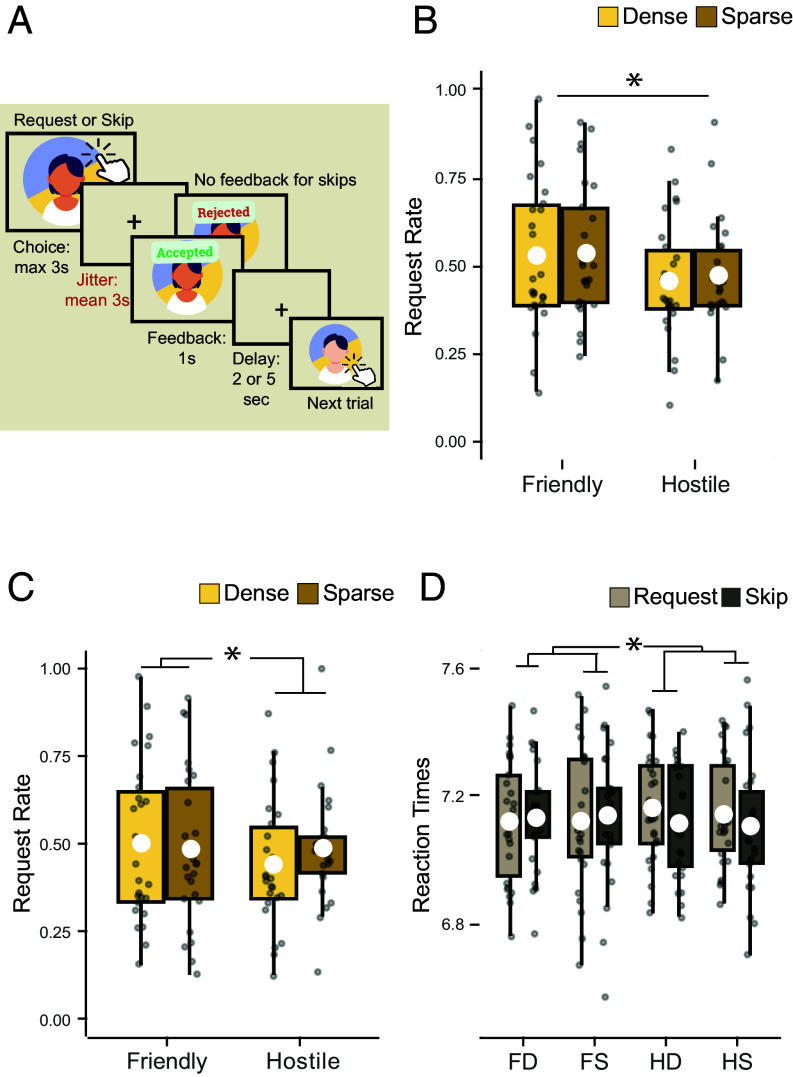
Behavioral results in the 7 T-fMRI cohort (n = 24). (*A*) Schematic representation of the Friend Request Task with slower timings optimized to capture slow BOLD signals. (*B*) Boxplots indicate the effect of background social environment on request rates in all trials (the white circle indicates mean; box boundaries indicate interquartile range [IQR], encompassing the middle 50% of the data; whiskers extend to furthest data points within 1.5 × IQR from the box boundaries). Request rates were higher in friendly blocks. The *y*-axis represents request rates, the *x*-axis represents friendliness levels, and color represents density levels. (*C*) Effect of background social environments on all but the first 10 trials in each block. Request rates were higher in friendly and sparse blocks. (*D*) effect of environments on RTs split by action type (request/skip). RTs were faster in friendly blocks, especially when the participant skipped sending a friend request. The *y*-axis represents RTs, the *x*-axis represents the four types of environments (FD: friendly, dense; FS: friendly, sparse; HD: hostile, dense; HS: hostile, sparse), and color represents participant action (request/skip).

In our third line of investigation, we turned to the neural underpinnings of affiliation choices. A smaller group of n = 24 participants played the same Friend Request Task as part of a high resolution (1.5 mm isotropic), rapid repetition time (1.96 s), accelerated ultra-high field (7 T) neuroimaging study. We took care to slightly adjust the task timings (Fig. 3*A*) to allow us to capture the slow blood oxygen level dependent (BOLD) response.

Before turning to the neural data, first, we checked whether participants in the fMRI experiment exhibited similar behavior in the Friend Request Task. As before, a 2 × 2 ANOVA showed a significant effect of friendliness on request rates (df = 25. F = 7.30, *P* = 0.012, $\eta_{p}^{2}$ = 0.226; Fig. 3*B*), suggesting that people were more likely to send a friend request in friendly environments compared to hostile environments. While there was no effect of density when examining all trials, when we carried out the same check of excluding the first 10 trials in each block that we had performed in the larger sample above, an interaction between friendliness and density was revealed (df = 25, F = 4.985, *P* = 0.035, $\eta_{p}^{2}$ = 0.166; Fig. 3*C*). This suggests participants needed an initial period in a task block to appreciate the density of the social environment they were currently in. This seemed particularly important in this slightly slowed-down version of the task used in the scanner.

Looking at the effects of environment statistics on participants’ choices to request or skip sending a friend request, a 2 × 2 × 2 ANOVA showed an interaction between friendliness and action on RT (df = 25, F = 6.836, *P* = 0.015, $\eta_{p}^{2}$ = 0.215; Fig. 3*D*). While the main effect of faster RTs in skip trials was not statistically significant in this smaller sample, consistent with the large online dataset, here again, people exhibited a tendency to be faster to skip than to send a request especially in hostile blocks (see *SI Appendix*, Fig. S4 for results that did replicate).

### Statistics Related to Social Environment Density Are Tracked in a Cortical–Subcortical Network.

Having confirmed that the behavioral results in the smaller MRI cohort of participants also reflected the social environment and largely confirmed our preregistered results from the larger online cohort, we next examined whether social background features might be reflected neurally in the BOLD response in a priori-defined ROIs. Even though this part of the analysis was not preregistered, we selected all five ROIs based on the literature before any data analysis. First, we selected the substantia nigra (SN) because tonic dopamine-linked midbrain nuclei like the SN in rats track opportunity costs associated with average reward rates (25). Additionally, midbrain dopaminergic regions in humans may represent social cravings akin to hunger (7). Second, we chose the anterior insula (aI) and habenula (Hb) because neural activity in this distributed network reflects the evaluation that a monetary opportunity has on behavior given the background context (21). Next, we selected the dorsal raphe nucleus (DRN) because other studies have emphasized the neuromodulatory system centered on the DRN in tracking the average value of the environment (9, 22) or transitions in its average value (23). As is the case for SN, DRN activity patterns also emerge in the context of interactions with aI and Hb (9, 22, 23, 26). And finally, we chose the dorsomedial prefrontal cortex (dmPFC) given its role in tracking and individuating social agents (31–39) [area 9 (24)].

**Fig. 4.**
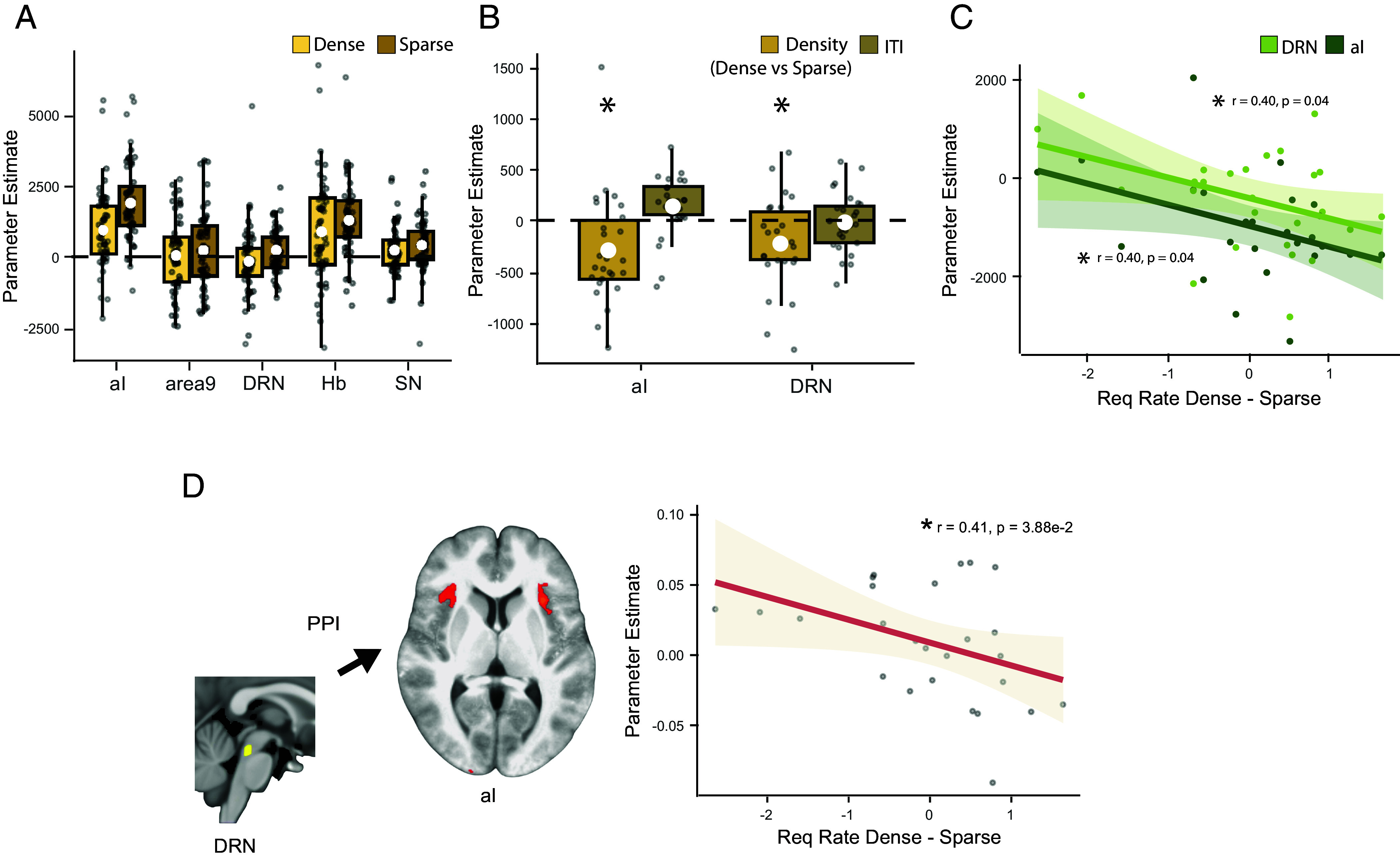
Neural effect of social density. (*A*) Boxplots indicate the effect of density of social opportunities across five regions of interest (ROIs). The *x*-axis denotes the ROI, the *y*-axis shows parameter estimates, separately for dense (yellow) and sparse (ochre) blocks (the white circle indicates mean; box boundaries indicate interquartile range [IQR], encompassing the middle 50% of the data; whiskers extend to furthest data points within 1.5 × IQR from the box boundaries). (*B*) In the aI and DRN, the two regions where density effects were significant in (*A*), density effects, shown as one bar reflecting dense vs. sparse (yellow), hold when controlling for local surprise by including past trial ITI (brown). Color represents explanatory variable from the GLM: yellow = parametric effect of density; brown = previous trial ITI. (*C*) Across participants, the size of the neural effect of density in aI and DRN relates to the size of the behavioral effect of density. The *x*-axis represents the behavioral effect of density calculated as the difference in request rates observed in dense versus sparse blocks. Color represents the parameter estimates obtained from DRN (light green) and aI (dark green). (*D*) Across participants, the PPI effect of density on aI–DRN interactions (PPI effect measured in the aI seeded at the DRN) was correlated with the behavioral effect of density.

We first examined BOLD responses related to the density of the social environment, or in other words, the frequency with which social affiliation opportunities arose. Parameter estimates were extracted from the five ROIs at the time of face onset, separately for all four types of environments. A mixed-model ANOVA showed a main effect of density of the social environment across ROIs (Fig. 4*A*). Sparser blocks were associated with increased BOLD activation at face onset than denser blocks (df =1, $\chi^{2}$ =7.97, *P* = 4.74e-3). There was also a density by region interaction (df = 4, $\chi^{2}$ = 10.96, *P* = 2.69e-2). Post hoc tests (corrected for multiple comparisons) revealed that the aI was the primary driver of this interaction; its density slope significantly differed from that of DRN (df = 900, t = 10.26, *P* < 1.0e-4), area 9 (df = 900, t = 9.55, *P* < 1.0e-4), SN (df = 900, t = 8.14, *P* < 1.0e-4), and showed a trend for the Hb (df=900, t = 2.43, *P* = 0.10). Region-wise post hoc tests employing a mixed model ANOVA (incorporating factors of both density and friendliness; friendliness effects are discussed below) showed a main effect of density in DRN (df = 1, $\chi^{2}$ = 7.65, *P* = 2.26e-2) and aI (df = 1, $\chi^{2}$ = 20.50, *P* = 2.98e-5) after correcting for multiple comparisons.

Given the main effect of density on neural activity was mainly driven by the DRN and aI, we further checked whether the effect of density truly reflected the extended experience of long intertrial intervals (ITIs), and thus the opportunities furnished by the social environment over the duration of the block, rather than simply the length of the most recent interval since encountering the last friend-seeking opportunity. If, instead, neural activity in DRN and aI was simply determined by the most recent ITI then it might be better interpreted in some other way, for example, potentially in relation to the next trial – the next face presentation – starting surprisingly early. We therefore assessed whether an effect of density was still present in these brain regions when controlling for the most recent intertrial interval (ITI), which served as a proxy for local trial-wise surprise. A linear mixed model showed that the density effect remained significant across both regions (df = 25, t = −2.19, *P* = 3.84e-2; Fig. 4*B*) even after including the most recent past ITI as additional regressor in the GLM. Further, a mixed model ANOVA showed that the density effect was significantly different from the past trial ITI effect (df = 1, $\chi^{2}$ = 14.37, *P* = 1.5e-4). Finally, we found that, across participants, the behavioral effect of density was correlated with the neural effect of density both in aI (df = 24, t = −2.13, r = 0.4, *P* = 4.34e-2; Fig. 4*C*) and DRN (df = 24, t = −2.12, r = 0.4, *P* = 4.42e-2). We, therefore, finally, examined whether interactions between DRN and aI also varied across participants in a manner that was related to variations in their behavior. A psychophysiological (PPI) analysis between DRN and aI, which was seeded at the DRN and predicted aI activity as a function of density and DRN activity, showed that the strength of density-dependent functional connectivity correlated with the behavioral effect of density on request rates (df = 24, r = −0.41, t = −2.18, *P* = 3.88e-2; Fig. 4*D*).

### Statistics Related to Social Environment Friendliness Are Tracked in a Similar Cortical–Subcortical Network.

**Fig. 5.**
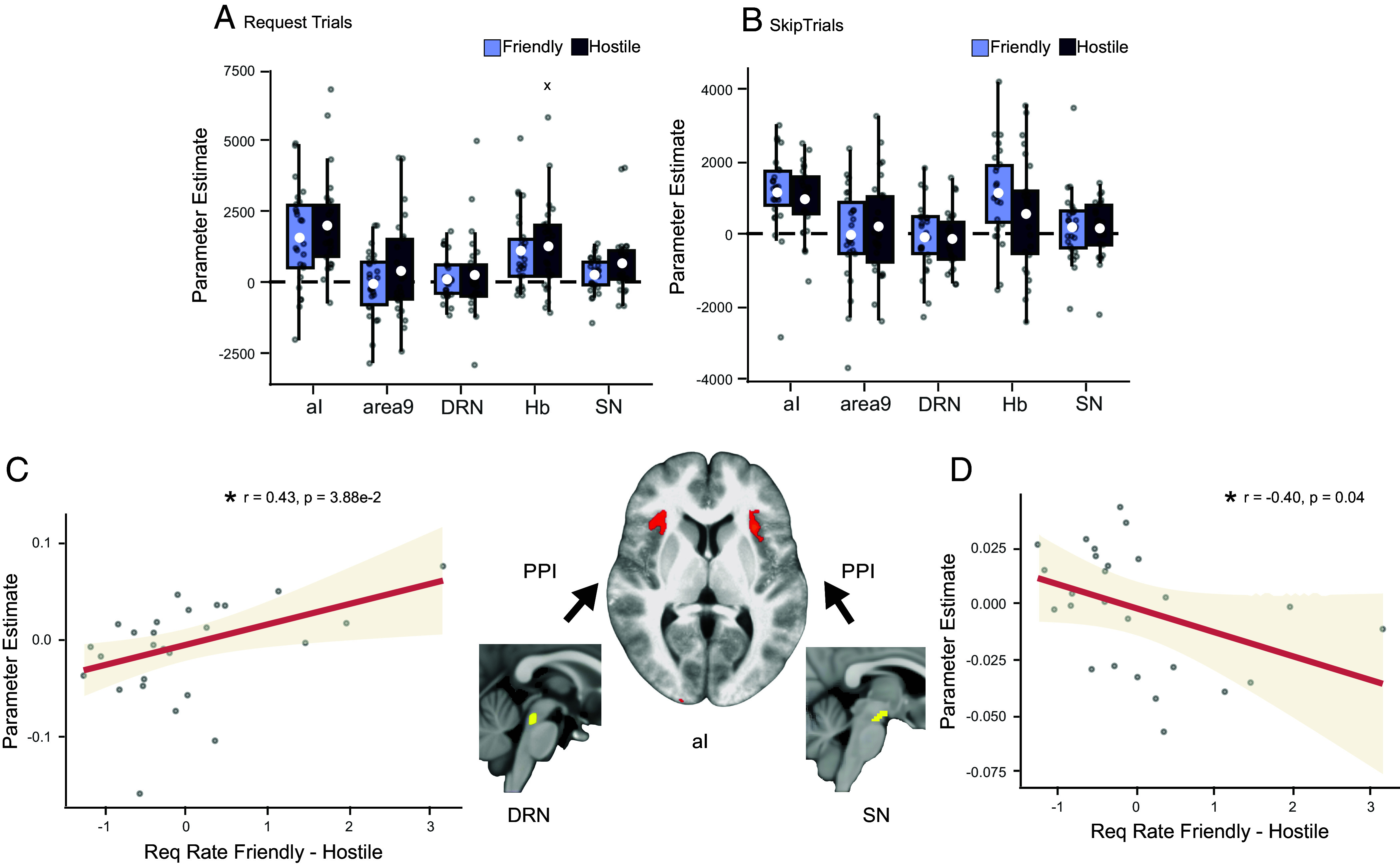
Neural effects of environment friendliness. (*A* and *B*) Boxplots indicate the friendliness effects in regions of interest (ROIs) separated by request and skip trials, at the time of face onset. The *x*-axis represents ROI, the *y*-axis represents parameter estimates, and color represents the levels of friendliness (the white circle indicates mean; box boundaries indicate interquartile range [IQR], encompassing the middle 50% of the data; whiskers extend to furthest data points within 1.5 × IQR from the box boundaries). BOLD activity differed between friendly and hostile blocks across ROIs, particularly when sending a friend request; hostile blocks led to greater BOLD responses to face onsets than friendly blocks. (*C*) Across participants, variation in interactions between DRN and aI as a function of friendliness (PPI effect measured in aI and seeded at the DRN) was related to variation in the behavioral effect of friendliness. (*D*) Across participants, variation in interactions between SN and aI as a function of friendliness (PPI between SN and aI as a function of the friendliness action interaction effect) was correlated with variation in the behavioral effect of friendliness.

In our second key line of investigation for the BOLD data, we then turned to potential effects of the friendliness of the environment, i.e. the likelihood of any given friendship request to be accepted. When we examined the effect of friendliness on neural activity, a mixed model ANOVA identified an interaction between friendliness and action across all areas (df = 1, $\chi^{2}$ = 8.62, *P* = 3.32e-3; Fig. 5 *A* and *B*). The effect of friendliness was stronger in request trials; in other words, BOLD activity differed between friendly and hostile blocks (with greater BOLD responses to face onset in hostile blocks), but it did so more in request trials.

Because we had found a relationship between social environment density and aI–DRN interactions in a PPI analysis, we carried out a similar analysis to assess whether DRN–aI interactions were also linked to the friendliness of a social environment. Again, variation in aI–DRN functional connectivity correlated with variation in the behavioral effect of friendliness on request rates (df = 24, r = 0.43, t = 2.37, *P* = 2.63e-2; Fig. 5*C*). However, because previous work (21) had particularly linked one of our other regions of interest, SN, and its interactions with aI, to action initiation, we used a second PPI analysis to examine the relationship between individual variation in SN-aI functional connectivity and individual variation in the impact that friendliness had on action (friend request or skip). SN-aI functional connectivity similarly correlated with the behavioral effect of friendliness on request rates (df = 24, r = −0.40, t = −2.15, *P* = 4.21e-2; Fig. 5*D*).

## Discussion

Both our own intuitions and a body of research evidence suggest that social connection depends on complex feelings of alignment in peoples’ personal views and perspectives, but also on simple factors such as the individuals’ proximity to and familiarity with one another (5). Here, we demonstrate that, in addition, the fundamental background statistics of social environments also impact participants’ propensities to initiate friendships. Using a unique task design that provided no incentives for or against sending friend requests, we found a significant effect of both the average friendliness of the social environment and the social density of the environment on affiliation request rates. Dense blocks had lower request rates compared to sparse blocks and friendly blocks had higher request rates compared to hostile blocks. The background environments also had a significant effect on RTs. Slower RTs were associated with sparser and friendlier environments. This result was robust across large discovery and replication cohorts and largely replicated in a smaller neuroimaging cohort.

Intriguingly, social affiliation initiation patterns resembled those seen during food-directed foraging (8), consistent with other recent studies that have observed parallels between social behavior and behaviors predicted by foraging theories originally constructed in the context of food seeking (40, 41). For example, birds allocate more resources to foraging in abundant environments (8) which is analogous to people sending more friend requests in friendly environments. Similarly, birds show higher selectivity when the encounter rate with the prey is high (15) and this is analogous to people showing lower request rates in denser environments. The behavioral parallels with foraging studies were also extended in our neural findings, where, for instance, sparser environments were shown to have stronger activations in the DRN, aI, as opposed to denser environments.

While drawing analogies between foraging and social affiliation decisions can help us make useful predictions about social behavior, the parallels with the procedures used in foraging studies are not entirely perfect. For instance, Krebs and colleagues (15) originally manipulated the value of each choice by offering birds different types of prey (some food items were small while others were large). Our task differs in that all faces carried the same reward value (either a request was accepted or rejected). In the future, we could attribute different values to faces to see how the relative frequencies of different values impact choices (22).

Recent studies have suggested that social connection may be represented similarly to other fundamental rewards such as food or money (7, 42). This view conceptualizes social affiliation as meeting a basic human need (6), analogous to how foraging behavior satisfies nutritional requirements. By paralleling established foraging paradigms, our study provides support for this framework by demonstrating that social reward-seeking follows similar behavioral patterns to those observed in nonsocial reward contexts.

However, our findings do not imply that social and nonsocial rewards are identical in every respect. Rather, our results suggest functional similarities in the behavioral strategies employed when seeking different types of rewards. Future research will need to directly contrast social and nonsocial rewards within the same experimental paradigm to determine whether these behavioral similarities reflect shared underlying mechanisms.

In our Friend Request Task, we chose a design that imposed no explicit monetary cost for sending a friend request to allow us to tap into people’s natural social tendencies in the absence of external incentives. Nevertheless, participants chose to skip sending friend requests about 50% of the time on average. A purely reward-driven model of decision making might predict that, in the absence of monetary costs, people would send a request to every person they encounter, maximizing both information gain and friendships. One explanation for the apparently “irrational” behavior observed is that while it is true that there was no explicit, quantified cost for sending a friend request, there was potentially an emotional cost in the form of a rejection, as well as an implicit effort cost, both of which might feel unpleasant. Thus, people might employ an implicit currency of emotional cost when considering making a friend request given that real-life experience indicates that it is emotionally challenging to tolerate rejections (43).

While we designed the Friend Request Task to examine core aspects of social decision‐making both inside and outside the scanner, a limitation is that this controlled paradigm cannot replicate the full complexity of real‐world friendship formation. Not surprisingly, our experiment, like many experiments in social cognitive neuroscience, omits many dynamic cues, reciprocal exchanges, and contextual nuances of real-life interactions and leaves open questions about how these mechanisms operate in real world environments. Future studies could address this gap by deploying higher‐level approaches—such as immersive virtual‐reality scenarios or in‐lab behavioral experiments with live interactions—to see how the patterns we observe scale up in ecological contexts.

In this experiment, we defined transdiagnostic mental health dimensions to create a simple yet robust profile of participant mental health and personality in a general population sample (44). Transdiagnostic definitions of mental illness challenge traditional disorder-based frameworks in favor of simple data-driven categories. In general, between-participant variation in the transdiagnostic factors we identified and focused on – Social Thriving, Sensation Seeking, Pleasure (reduced anhedonia) – were related to between-participant variation in one of the Friend Request Task’s simplest parameters – the total requests sent. The relationship between total requests and Social Thriving was mediated by sensation seeking. Such findings suggest both that forming friendships might be part of a larger drive to accumulate sensations or experiences and that variation in these propensities might be captured by a simple laboratory task. Thinking of social affiliation as part of a larger drive is consistent with results suggesting hunger for food and hunger for social contact have shared representations in the brain (7) and that social animals like macaques trade juice rewards in exchange for the opportunity to gaze at other monkeys (42).

The transdiagnostic Pleasure factor that we identified was especially intriguing. Not only was it related to the total friend requests that participants made but it was also related to the way in which the social environment impacted the sending of friend requests; people who had a higher pleasure score were also more sensitive to the average friendliness of the social environment and showed stronger adaptation of their friend request tendencies to the environment’s friendliness. In other words, they send relatively fewer requests in hostile and relatively more requests in friendly environments than the average person. One possible interpretation of this effect is that individuals with a greater experience of pleasure also have increased sensitivity to potential rejection, causing them to adjust their friend‐request rates more markedly in response to the social environment’s friendliness.

Notably, the Pleasure factor included loadings from the apathy motivation index. Given theoretical models linking apathy to the dopaminergic system (25, 45), the pleasure-friendliness link suggested that background friendliness and its interaction with action would be encoded in midbrain dopaminergic areas like the SN. This proved to be the case.

One open question relates to whether participants treated our task akin to a real social situation, despite its simple structure and virtual nature. While a simplified task like the one used here, like many experimental tasks in social psychology and cognitive neuroscience, can clearly not capture the full reciprocity, ongoing investment, or rich sensory context of real-world friendship formation, several key aspects of our findings suggest that participants were sensitive to its social nature.

First, participant friendship requests were linked to transdiagnostic “social thriving” factor—which loaded heavily on loneliness and real‐world network size—suggesting that requests reflected genuine affiliation motives. Second, if people were simply treating an acceptance as reward, but were lacking any sensitivity to rejection (i.e., treating rejection as a net zero increment, with no social cost attached), we would expect participants to send a request on every trial. However, as discussed earlier, participants sent a request on about half of the trials, and fewer in hostile environments, suggesting a sensitivity to rejections which only have a social meaning. Finally, the number of “friends” already accumulated reduced the likelihood of sending a subsequent friend request (exploratory analysis described in *SI Appendix*) —consistent with real‐world friendship dynamics, rather than a “more is always better” paradigm likely associated with accumulating likes or followers in a more artificial or “social media” like setting. Nevertheless, we acknowledge that the process that we study is a simple one that may be part of many other social affiliative behaviors alongside friendship seeking.

In a 7 T fMRI study, we found that background statistics of social environments were tracked by the brain. A priori ROIs—namely the DRN, dmPFC area 9, aI, Hb, SN—encoded opportunities for friendships differently depending on environmental density; that is, at face onset, the activity in these ROIs reflected how often opportunities for affiliation arose. Further, this effect was present even when controlling for the last ITI, ruling out a purely local density code or an interpretation in terms of activity simply reflecting some form of surprise, such as a surprisingly early trial onset. The effect was strongest in DRN and aI. Indeed, a PPI analysis showed that DRN and aI connectivity varied across individuals as a function of the behavioral effect of density suggesting an intimate link between DRN–aI connectivity and mediation of the social density effect. DRN, aI, and DRN–aI connectivity have recently been implicated in tracking the average value of food, monetary rewards, and the potential costs of threats in the environment in macaques and humans (9, 22, 23, 26). The present results suggest related roles in tracking the statistics of social environments. Importantly, in other tasks in which activity in DRN and interconnected areas has been identified, we have found that administration of selective serotonin reuptake inhibitors (SSRIs) – one of the most commonly used antidepressants – leads to changes in behavior (22). SSRIs are thought to exert their effects partly by moderating DRN’s influence over activity elsewhere in the brain.

The brain regions we investigated also encoded an interaction between friendliness and action. A PPI analysis showed that the interaction between friendliness and action (send versus skip friend request) covaried as a function of connectivity between SN and aI. SN and aI–SN interactions have recently been implicated in the integration of opportunity and background value and their translation into action initiation (18, 21). This is consistent with the view that social rewards might be represented in a similar manner in the brain as monetary or gustatory rewards (46). While the SN is a key source of dopamine, a direct manipulation of dopamine levels, for instance using a dopamine antagonist, might provide insight into the role of dopaminergic neurons in tracking the friendliness of environments.

## Methods

### Experiment 1: Large-Scale Behavioral Investigation of Friend Request Seeking and Relationships with Individual Differences in Mental Health Dimensions.

#### Participants, task and questionnaires.

Ethical approval for this study was obtained through the Medical Sciences Interdivisional Research Ethics Committee (MS-IDREC; ref: R73912/RE001). Informed consent was obtained from each participant before they began the experiment. Multiple datasets were collected using Prolific (prolific.co) as part of Experiment 1, split into a discovery and confirmatory dataset. For the confirmatory dataset, we collected data from 1,018 participants out of which 783 met our inclusion criteria (mean age = 27.3, males = 392, females = 380, other = 11; see *SI Appendix* for details on the discovery sample and our inclusion criteria).

In the task, participants were asked to imagine moving to a new city and making new friends. In each of four different block types (dense/sparse x friendly/hostile), participants were shown a series of faces and given the opportunity to either send a friend request or skip the opportunity. If participants sent a friend request, the next screen would show them whether their request was accepted or rejected (for more task details, see *SI Appendix*).

In addition to the behavioral task, participants completed a series of standardized questionnaires to assess their personality and psychiatric profile in both social and nonsocial domains (for a complete list, see *SI Appendix*).

#### Statistical analysis.

Data were analyzed using R v4.3.3 (47) running on RStudio v2023.12 (48). Figures were assembled and prepared for publication using Canva (https://www.canva.com) and Adobe Illustrator v28.4.1 (https://adobe.com). All binary categorical variables were coded as -1 and 1. RTs were log transformed. A 2 × 2 repeated measured ANOVA was used to examine the effect of friendliness and density on request rates and RTs. A 2 × 2 × 2 repeated ANOVA was used to examine the effects of friendliness and density when RTs were further split by action (requests or skips). A similar 2 × 2 × 2 ANOVA was used to examine the effects of past trial feedback, friendliness, and density on request rates. Since all our independent variables only ever had two levels, it was not possible to violate the assumption of sphericity. Significance threshold for all tests were set at *P* = 0.05.

For the factor analysis, a scree test was used to determine the number of factors, and the factors were obtained using a promax rotation and minimum residual factoring method. The scores were extracted using the Thurnstone method (49). A Pearson’s correlation was used to assess the strength of the association between these factors and behavioral measures (Fig. 2). A Sobel test was used to carry out mediation analysis (for further details, see *SI Appendix*).

All hypotheses were preregistered (osf.io/62hw7) and are described in the *SI Appendix*.

### Experiment 2: 7 T High-Resolution MRI Study of Friend Request Task.

#### Participants, task and behavioral analysis.

The study was approved by the Medical Sciences Division Interdivisional Research Ethics Committee (Ethics Approval Reference: R77443/RE002). A total of 30 participants were recruited and gave informed consent, out of which 26 (mean age = 24.4, males = 10, females = 16) participants met the inclusion criteria (*SI Appendix*). The task was identical to Experiment 1 apart from small adjustments to the timing (Fig. 3*A* and *SI Appendix*). As with the online behavioral task, effect of friendliness and density on request rates and RTs were analyzed using a 2 × 2 repeated measures ANOVA (Fig. 3 *B* and *C*). Trials were further split into requests and skips, and a 2 × 2 × 2 ANOVA was used to measure the effect of friendliness, density, and action (request versus skip) on RTs (Fig. 3*D*).

#### MRI data acquisition and analysis.

High-resolution MRI data including a T1, functional scans (~45 min), and a fieldmap were acquired on a 7 T Siemens scanner (see *SI Appendix* for sequence details).

FMRI data were preprocessed and analyzed using FMRIB’s Software Library (50) (FSL), Python (51), and the package fslpy to interface with FSL (52). Functional images were converted from dicom to nifti and then reoriented to standard orientation. Structural images were bias-corrected using FSL’s anatomical preprocessing script fsl_anat. Brain extraction was performed using SynthStrip (53). Functional images were preprocessed using motion correction, fieldmap unwarping, highpass filtering, and registration to standard space (see *SI Appendix* for more details). Following preprocessing, a General Linear Model (GLM) containing 16 task-based regressors (face onset, parametric friendliness, parametric density, and parametric action for each of four block types) plus motion and physiological noise regressors was estimated using FMRIB’s Expert Analysis Tool (54) (FEAT v6; see *SI Appendix*).

The parameter estimates obtained from fitting the GLM were then extracted for five predefined regions of interest (ROI): DRN, Hb, SN, aI, dlPFC. While the neural regions of interest were not formally preregistered, we drew up a list of the relevant ROIs based on a review of the relevant foraging literature (10, 18, 21, 22, 26) prior to any data analysis. For all ROIs but the DRN, masks were obtained from similar previous studies (26) and standard atlases (55). The DRN mask was drawn using the standard diffusion template (FMRIB58_FA) included as part of FSL (*SI Appendix*, Fig. S5).

A linear mixed-model (LMM) was used to evaluate the effect of friendliness and density in these five predefined ROIs (Figs. 4 and 5). The LMM included friendliness, density, action, region, and their interactions as fixed effects, and friendliness, density, and action as random effects that varied across participants. The significance of the fixed effects and their interactions was evaluated using a mixed-model ANOVA implementing type II Wald chi-square tests.

Among the regions that showed the density effect, we tested whether the neural effect was related to the behavioral effect using a Pearson’s correlation. We also tested whether density effects were truly global contextual effects of density, or simply a consequence of the previous ITI as a proxy for local density or surprise. A LMM was also used on the parameter estimates obtained from a modified GLM to test for global effects of density over and above previous ITI effects (*SI Appendix*, Fig. S4*B*). Region was included as a fixed effect, and a random intercept was included to account for variability between subjects. A *t* test using Satterthwaite’s method was used to assess statistical significance of fixed effects. A mixed model ANOVA was used to determine whether the parameter estimates for density and past trial ITI differed from each other. Finally, a psychophysiological interaction (PPI) analysis was performed, seeded at the DRN, to test for changes in functional connectivity between DRN and aI as a function of density and friendliness (*SI Appendix*, Figs. S4*D* and S5*C*).

## Supplementary Material

Appendix 01 (PDF)

## Data Availability

The data reported in the online behavioral experiment as well as the unthresholded statistical group maps of the fMRI experiment are available at the project’s OSF directory (https://osf.io/jf6vs/) (56). All data used in the HCP study (mentioned in the *SI Appendix*) are available for download from the Human Connectome Project (www.humanconnectome.org) (57). Users must apply for access and agree to the HCP data use terms (for details, see https://www.humanconnectome.org/study/hcp-young-adult/data-use-terms). Here, we used both Open Access and Restricted data. Code used to analyse behavioural and neural data is available at the project’s OSF directory (https://osf.io/jf6vs/) (56).
