# Supplementary material for: Fundamental features of social environments determine rate of social affiliation
Source: Proc Natl Acad Sci U S A. 2025 Oct 14;122(42):e2506243122. doi: 10.1073/pnas.2506243122 (PMC12557543; doi:10.1073/pnas.2506243122)
Supplement: Supplementary file 1 — Appendix 01 (PDF) [file pnas.2506243122.sapp.pdf]

Supplementary Information for Fundamental Features of Social Environments Determine Rate of Social Affiliation

Sankalp Garud[a+], Miruna Rascu[a], Sorcha Hamilton[ac], Ingrid Yu[a], Matthew F.S. Rushworth[a]*, and Miriam C. Klein-Flügge[ab]*
*Shared senior authors

[a]Wellcome Centre for Integrative Neuroimaging, Department of Experimental Psychology, University of Oxford, Oxford OX1 3SR, United Kingdom
[b]Department of Psychiatry, Warneford Hospital, University of Oxford, Oxford OX3 7JX, United Kingdom
[c]Department of Psychology, University of Bath, Bath BA2 7AY, United Kingdom

Corresponding author: Sankalp Garud (sankalp.garud@ndcn.ox.ac.uk)

Additional methods and results (online and MR cohorts)

1. **Detailed Methods**

Experiment 1: Large-scale behavioural investigation of friend request seeking and relationships with individual differences in mental health dimensions

Participants

The initial discovery sample included 300 healthy participants out of which 218 participants (mean age = 25.3, males =110, females = 107, other = 1) met the inclusion criteria. For the confirmatory dataset, an a priori power analysis was conducted using G*Power version 3.1[1] for sample size estimation, based on data from our discovery sample. The smallest size of an effect of interest in the discovery study was 0.017. With a significance criterion of α = .05 and power = .95, the minimum sample size needed would be N = 767 for a t-test computed using the Pearsons correlation coefficient. Thus, the power analysis suggested collecting a minimum sample of size 997 after accounting for a 30% attrition rate.

Only participants aged between 18 and 40 years old and who had normal or corrected to normal vision were included in the study.

After collecting pseudonymised data through Prolific, the data were checked for completion. Participants who completed the task were paid at a rate of £6/hr for their participation and they received an additional £2 bonus for task completion. All completed datasets were further examined based on the following preregistered exclusion criteria developed using the discovery sample.

1. Total timeouts (trials in which participants failed to respond after 3 seconds) exceeded 15 in number
2. Request rate per block was either 1 or 0, and that happened for 2 or more blocks out of the total 12.
3. Participants failed to answer repeat questions (a selection of five questions from five different questionnaires presented again towards the end of the experiment) within a 2-point absolute deviation, and this happened for two or more items.
4. Maximum standard deviation of request rates within the same block type was greater than 0.3, suggesting an inconsistent or random choice strategy.
5. Task effects were outside 3 standard deviations of their sample means.

Task

A computer-based task was coded using jsPsych[2] and was uploaded to the Department of Experimental Psychology's server through "Just Another Tool for Online Studies"[3] (JATOS) and Pavlovia (pavlovia.org).

In the instructions prior to starting the task, participants were given a cover story in which they were asked to imagine moving to a new city and making new friends. To help them create connections, they would be taken through different clubs which differed in two respects: the friendliness of the people in the club and the number of people that appeared in the club. In each club, they would make repeated choices to send friendship requests to individuals in that club, or to not send a request and skip an opportunity to make a new friend.

Four combinations of clubs were possible: friendly-dense (FD), friendly-sparse (FS), hostile-dense (HD), and hostile-sparse (HS) (Fig.1b). In a friendly club, 80% of all friend requests that a participant sent were accepted whereas in a hostile club, only 20% of all requests were accepted. In a dense club, the time between two consecutive encounters with other club members (being shown a club member face) was briefer; the faces appeared every 2 s with a jitter between 400 and 700 ms. In a sparse club, the time between two consecutive faces was 5 s with an equivalent jitter. On average, a participant saw approximately 32 faces in the dense environments and approximately 19 in the sparse environments.

The background colour of the club (blue or green) indicated the level of friendliness of the club and the background pattern (smaller or larger circles) indicated the level of density of the club. For instance, a blue background with small circles might have indicated a friendly-dense block for some participants. The mapping of backgrounds and patterns to club-type was counterbalanced across participants. At the start of each club, participants were informed about the type of club they would be entering.

Inside each club, participants were shown a series of faces expressing a neutral emotion and given the opportunity to send friend requests. The faces presented as stimuli were obtained from the Chicago Faces Database[4]. The order of the faces was randomised between participants. Faces were sampled at random, and participants saw each face only once.

For every face shown on the screen, participants could either choose to send a friend request by pressing 'j' on their keyboard or they could choose to skip by pressing 'k'. Each face would stay on the screen for as long as the participants took to respond, or for a maximum time of 3 s. If participants sent a friend request, the next screen would show them whether their request was accepted or rejected. This feedback appeared as text over the face and was shown for a duration of 1 s (see Fig.1a for an illustration of the trial structure). If they chose to skip, then a fixation cross of the same duration would appear on the screen. If they failed to respond within the stipulated window, a warning sign appeared on screen indicating they were too slow to respond.

Each block (or club) lasted for a duration of 2.5 minutes. Participants were shown a circular timer on the top right of the screen which indicated the elapsed time. The entire experiment comprised a total of 12 blocks, with all 4 types of blocks appearing three times. The order in which participants encountered these blocks was randomised.

Before the main task began, participants were given a chance to play through one practice block to help them familiarise themselves with the design and controls. After they completed the practice block, their understanding of the task was tested with a short quiz comprising five questions. They were given two attempts to get all questions correct.

During piloting, we noticed that people might not be paying attention to the immediate friendliness of the environment. To make the friendliness salient, we added an attention check. We asked participants “Did the previous person accept or reject your request” roughly every 20 seconds. Participants indicated their responses by pressing ‘f’ on the computer keyboard if accepted, ‘g’ if rejected, and ‘h’ if they skipped the request. This version was used as the task in the discovery dataset. The fact that we observed an effect of friendliness and density of the social environments on acceptance rates suggests that the final task design was sufficiently engaging for participants

Questionnaires

In addition to the behavioural task, participants completed a series of standardised questionnaires to assess their personality and psychiatric profile. Each questionnaire had a lower time limit of 2 s per question to ensure participants took adequate time to understand the questions. If participants completed an entire questionnaire before this lower time limit (2 s x the number of questions), they were given a warning that asked them to reconsider their responses.

We asked participants to complete a set of both social and non-social questionnaires. Scales with predefined subscales were segmented as specified; for questionnaires without pre-defined subscales, we performed a factor analysis on the individual items in the discovery sample to derive their subscales. For obtaining social measures, we used the Lubben Social Network Scale (LSNS) to measure objective social network size[5]. The LSNS comprises two subscales: Friends and Family. Next, we included the UCLA Loneliness Scale Version 3 (UCLA) to measure people’s subjective sense of loneliness[6]. This scale comprises Thriving, Lack of Connection, and Basic Connection as its three subscales. In addition, we included the Social Connectedness Scale (SCS) to measure participants degree of feeling socially connected[7]. The SCS breaks down into Social Assurance and Connectedness subscales. We also used the Relationship Questionnaire (RQ) to measure participants attachment styles[8]. The RQ comprises subscales measuring four attachment styles: Secure, Preoccupied, Fearful Avoidant, Dismissing Avoidant. We also used the Social Pain Questionnaire[9] (SPQ). Finally, we used the anxiety subscale of the Liebowitz Social Anxiety Scale[10] (LSA).

Social well-being is also impaired in general mental health conditions like depression and anxiety. To capture such non-social dimensions of well-being, we included the Apathy Motivation Index (AMI) to measure apathy[11]. The AMI comprises Social, Emotional, and Behavioural subscales. In addition, we included Beck’s Depression Inventory[12] (BDI) and the Snaith-Hamilton Pleasure Score[13] (SHAPS) to measure depression and its associated symptom of anhedonia. The BDI comprises Somatic, Cognitive, and Affective subscales[14], whereas the SHAPS comprises Sensory, Personal, and Other-related types of pleasure. We also included the Rosenberg Self-Esteem scale[15] (RSE) to measure self-esteem and the revised Learned Helplessness Scale[16] (LHS) to measure learned helplessness. The LHS breaks down into Perseverance, Confidence, and Helplessness subscales.

We included some control questionnaires to assess the specificity of any identified relationships with social or psychiatric dimensions. We acquired data related to symptoms of obsession and compulsion using the checking and neutralising subscales of the Obsessive Compulsive Inventory revised[17] (OCI). We also recorded impulsivity using the Urgency, Premeditation (lack of), Perseverance (lack of), Sensation Seeking, Positive Urgency, Impulsive Behavior Scale[18] – short (UPPSP), and anxiety using the State-Trait Inventory of Cognitive and Somatic Anxiety[19] (STICSA).

The UPPSP has Sensation Seeking, Positive Urgency, Negative Urgency, Lack of Premeditation, and Lack of Perseverance as its subscales. Likewise, the STICSA consists of Somatic and Cognitive subscales. Finally, we included the Pain Sensitivity Questionnaire[20] (PSQ).

Questionnaires were presented in two sets. The first set, comprising SHAPS, UCLA, BDI, LHS, AQ, RQ, PSQ, SPQ, was presented before the behavioural task commenced. The second set, comprising SCS, OCI, LSNS, AMI, UPPSP, LSA, STICSA, was presented after the task ended. In addition, five pre-selected items from these questionnaires were presented again at the end of the study with the intention of using them for data quality checks.

Pre-registered hypothesis
We pre-registered the following hypotheses for main task effects (osf.io/62hw7):

(a) People would send friend requests at a higher rate (where request rate is defined as the percentage of trials, out of all trials, on which a request was sent) and will be slower to respond in friendly compared to hostile environments.

(b) People would send friend request at a higher rate and will be slower to respond in sparse compared to dense environments.

(c) People would send friend requests at a higher rate after acceptance than they will after rejections.

(d) People would send friend requests at a higher rate following a previous request particularly in dense environments but will be more likely to make requests following a skip (a non-request) in sparse environments.

(e) People would be faster to skip than to request overall. However, this difference between reaction times will be higher in hostile environments than in friendly environments. Similarly, the difference will be higher in dense environments as compared to sparse ones

Distinct factors from personality and mental health questionnaires should be identifiable, that load onto behaviours related to:

- Social Thriving
- Obsession/Compulsion
- Social Pain
- Sensation seeking/Urgency
- Pleasure
- Depression-Anxiety
- Impulsivity

In addition, individual variation in these factors should be predicted by individual variation in task measures. More specifically:

Total requests should relate to factors of social thriving and social pain in the following directions:
- Positively to social thriving
- Negatively to social pain

Total requests should relate to non-social factors in the following manner:
- Positively to sensation seeking
- Positively to pleasure

In a discovery sample, we found that social thriving and sensation seeking were correlated. We then hypothesised that sensation seeking would mediate the relationship between social thriving and total requests. we found a trend (p=0.08) for this mediation using a Sobel test. With a larger sample size, we expected this relationship to hold true. So, we predicted that:

• Sensation seeking would mediate the effect of social thriving on total requests.
• Next, the density effect on reaction times would relate:
• Positively to social thriving
• Positively to sensation seeking

As for the previous hypothesis, we expected the relationship between the density effect on reaction times and social thriving to be mediated by sensation seeking. Finally, the friendliness effect on choices to request or skip social engagement was expected to relate:

• Positively to pleasure

Statistical Analysis

To test the robustness of our findings, we conducted an additional exploratory analysis excluding the first 10 trials of each block. This analysis addressed two potential concerns: (1) that participants might experience reduced task engagement or forget instructions over time, and (2) that while participants received explicit information about block density ('dense' or 'sparse'), they were not provided with detailed breakdowns of the inter-trial interval distributions and might need several trials to update their estimates of the actual timing parameters. By omitting the initial trials, we could assess whether behavioural patterns remained consistent after participants had direct experience with the precise timing and frequency characteristics of each environment. This exclusion analysis yielded results consistent with our primary analyses that included all trials, confirming that our effects are robust both from initial block entry and throughout sustained task performance.

Some questionnaires did not come with predefined subscales. For such questionnaires, we undertook factor analysis and defined data-driven subscales. These questionnaires included the SHAPS, which decomposed into sensory, personal, and other-related types of pleasure subscales, UCLA, which decomposed into thriving, lack of connection, and basic connection subscales, and the LHS, which decomposed into perseverance, confidence, and helplessness subscales. Multiple comparisons were not corrected for as the tests were pre-registered.

Experiment 2: 7T high-resolution MRI study of Friend Request Task

Participants
Participants were recruited from the Oxford area using the Oxford Participant Recruitment system and other online and email-based advertisement platforms. Prior to attending the scan, participants were screened on the telephone using a standardised Wellcome Centre for Integrative Neuroimaging (WIN) 7T MRI screening form to ensure that they were eligible and safe to be scanned.

We recruited 18–40-year-old right-handed healthy individuals with normal or corrected to normal vision who were fluent in English and 7T-safe. Informed consent was obtained from all participants before the study commenced. Prior to entering the scanner, participants were asked

to read through a slideshow which presented instructions about the task and the MRI scanning environment. Participants then changed into scrubs and were screened by a trained radiographer.

Behavioural exclusion criteria were similar as those preregistered for the online study (the criteria for the online study were stricter to account for noisy behaviour and questionnaire-related checks). As a result, three participants were excluded because their request rates were either 0 or 1 for two or more blocks. One further participant was excluded because the standard deviation in their request rates exceeded 0.3. The final sample, therefore, comprised 26 participants.

Task

While in the scanner, participants played the Friend Request Task described above, albeit with some modifications to make it possible to obtain reliable estimates of brain activity at specific points in each trial given the slow haemodynamic response function (HRF). First, an additional jittered delay (sampled from a gamma distribution centred at 3 s) was added between the response and the outcome to allow dissociation of the HRFs associated with these two events. Next, the block duration was increased from 2.5 minutes to 3 minutes to compensate for the trials that would otherwise have been lost after adding the aforementioned delay (Fig. 3a).

Participants indicated their responses by using a button box with two buttons. Participants were instructed to place their index finger over the first button and their middle finger over the second button. The first button was used to send a friend request, whereas the second button was used to skip the opportunity to send a friend request. This button box was placed in the right hand of the participants as they lay inside the scanner. Similarly to the online task, each participant played the task for 12 blocks, with three blocks each of the four different types of environments. The entire task lasted for approximately 45 minutes.

ROIs

We focused on a cortico-subcortical circuit implicated in the tracking of the background statistics of reward environments during foraging[21–26,26–31] to test whether they played an analogous role in social affiliation seeking. We investigated activity across a distributed cortico-subocortical circuit comprising DRN, SN, Hb, dmPFC and aI,

MRI protocol

Scans were acquired on a 7T Siemens scanner. Structural scans were obtained using a T1-weighted sequence with a 0.7 mm isometric resolution; GeneRalized Autocalibrating Partial Parallel Acquisition (GRAPPA) acceleration factor of 2; TR 2.2 ms; TE 3.02 ms; 256 slices. The total duration of the structural scan was 6 minutes 35 s. Following the T1 structural scan, field-map images were acquired using a 2 mm isometric resolution; TR 620 ms; TE1 4.08 ms; TE2 5.1 ms; 73 slices; -30-degree angle. The total time to acquire the fieldmap was 2 minutes 2 seconds. Functional scans were then collected while participants performed the Friend Request Task. A multiband sequence was used to obtain a total of 74 slices tilted at a -30-degree angle with a 1.5mm isometric resolution; multiband acceleration factor 2; GRAPPA acceleration factor 2; TR 2.033 s, TE 18.4 ms. The total duration differed slightly between participants depending on their block breaks but was on average 46 minutes 45 seconds. Respiration and heart rate data were acquired while the participants lay in the scanner using instruments manufactured by BIOPAC systems. The total session duration, including set up time, was approximately 75 minutes.

Preprocessing

Fieldmap unwarping was performed using a spatial smoothing parameter set at 3mm of full width half maximum (FWHM). Highpass temporal filtering was also applied. Functional images were registered from their native space to structural space using FMRIB's Linear Image Registration Tool (FLIRT), and further registered to standard space using FMRIB's Nonlinear Image Registration Tool[32] (FNIRT). The Montreal Neurological Institute (MNI) 152 image in 1mm resolution was used as a standard brain template.

GLM

A double gamma haemodynamic response function was used to predict Blood Oxygen Level Dependent (BOLD) responses from task events[33]. The following task based regressors were used in the first GLM (GLM1). Our GLM included 16 regressors total. For request trials, we modeled three regressors per block type: (1) face onsets, (2) outcome onsets, and (3) parametric outcomes (accept/reject), yielding 12 regressors across four block types. For skip trials, we modelled one regressor per block type: (1) face onsets (no outcome-related regressors since skip trials lack outcomes), yielding 4 regressors across four block types. All parametric regressors were z-scored. In addition, six basic motion regressors and 33 physiological noise regressors (cosine and sine of basic cardiac and respiratory regressors modelled with an order of 4, and thus 16 regressors; multiplicative cardiac and respiratory terms cos(c + r), sin(c + r), cos(c − r), sin(c − r), each modelled using an order of 2, and thus again 16 regressors; plus respiration volume per time) were included in the GLM. Physiological noise correction was performed using physiological noise modelling (PNM) tool (https://fsl.fmrib.ox.ac.uk/fsl/fslwiki/PNM), part of the FSL package[34].

To test for global effects of density above and beyond previous ITI effects, we extended GLM1 to GLM2 which contained identical regressors, but which did not separate them by block-type or action and included a past trial ITI parametric regressor. Thus, GLM2 included five regressors in total, four regressors (face onset, parametric density, parametric friendliness, parametric action) and a new fifth regressor capturing past trial ITI.

A third GLM was used with face onsets, friendliness, density, action, the DRN time course, and the PPI regressor, which was the interaction between the time course (physiological regressor) of DRN activity and density (psychological regressor). Average parameter estimates were then extracted for the PPI regressor from the aI. A Pearson's correlation was used to determine whether individual variation in the strength of the PPI estimates was related to individual variation in the behavioural effect of density. An equivalent PPI analysis was performed seeded at the SN to test for functional connectivity with the aI that co-varied as a function of the interaction between friendliness and action (Fig 5d). A recent study highlighted the importance of ensuring time-related confounds do not influence PPI results[35], which we ensured by temporally counterbalancing all block types.

Experiment 3: Large-scale neuroimaging relationships between anhedonia/pleasure and network resting connectivity in the SN-aI network

Model fitting

Two datasets were used in this study. First, for model evaluation and fitting, data from the replication cohort in Experiment 1 were used. Next, for testing the relationship between psychiatric factors and resting-state functional connectivity in the network discovered in Experiment 2, we used the Human Connectome Project (HCP) young-adult database. For this, data and ethics were provided by the WU-Minn Consortium (Principal Investigators: David Van Essen and Kamil Ugurbil; 1U54MH091657; https://www.humanconnectome.org), funded by the

16 NIH Institutes and Centres that support the NIH Blueprint for Neuroscience Research and by the McDonnell Centre for Systems Neuroscience at Washington University. All participants gave informed consent and were reimbursed for their time ($450 for 3T MRI) and travel. HCP participants were scanned at the McDonnell Centre for Systems Neuroscience at Washington University, University of Minnesota (WU-Minn), USA, on a Siemens Skyra 3 Tesla scanner.

First, we tried to establish a relationship between HCP participants' mental health dimensions and those identified in Experiment 1, such as pleasure/anhedonia. To do so, we asked participants from our study in Experiment 1 to complete some of the same questionnaires that participants from the HCP dataset had completed as well. These overlapping questionnaires meant we had some comparable data in both Experiment 1's large online cohort and the HCP participants. This questionnaire data formed the basis for the first step of our analyses here. We then used machine learning techniques to establish a correspondence between the questionnaire variables we had studied in Experiment 1 and the questionnaire variables obtained in the HCP study.

The questionnaires available in both cohorts that we used were the NIH friendship and emotional support toolboxes[36] and the Ten item personality inventory (TIPI), comprising the agreeableness, openness to experience, emotional stability, extraversion, and conscientiousness sub-scales[37]. These questionnaires were thought to have the best chance (amongst questionnaires available as part of the HCP dataset) of describing variance related to the social and non-social features that we had found to bear relationships with performance on the Friend Request Task.

For model fitting, first, questionnaire scores were scaled and centred (z-scored) across participants. Then, the dataset was split into training and testing datasets (three quarters of the data were used for training and the remaining quarter was used for testing).

Four types of models were used to predict factor scores from the common set of questionnaires mentioned above: neural networks (nnet), simple linear regression (lm), linear regression with penalised maximum likehood (lmnet), and random forests (rf).

Hyperparameters for the models were tuned using the 'tuning' package in R. Models were then compared using the root mean squared error (rsme) and r^2 metrics. Winning models selected were those that minimised rsme. Finally, the winning models were validated on the testing dataset using a Pearson's correlation between the true and predicted values. Model predictions for the Pleasure (or reduced anhedonia) score were obtained for all participants using the winning model.

Resting state fMRI data
Each run lasted 14.4 minutes, had a repetition time (TR) of 720ms, echo time (TE) of 33ms, resolution of 2mm isotropic. A total of 72 slices were acquired with a multiband factor of 8 resulting in 1200 timepoints. These data were corrected for distortions, temporally-filtered, minimally smoothed and projected onto a surface reconstruction obtained from aT1-weighted image.

2. Additional results: Logistic regression mixed-effects model for choices

Here we report all equivalent tests shown in the main part of the manuscript using mixed models.

A logistic regression mixed model ANOVA estimated on the behavioural data of the 783 participants from the online cohort showed similar effects for friendliness and density on choices as reported in the main text. The main effects of friendliness (df=1, $\chi^2 = 82.24$, p < 2.2e-16) and density (df=1, χ^2=164.38, p<2.2e-16) were significant. The interaction between friendliness and density revealed a trend (df=1, χ^2=3.09, p=0.08). Further, the interaction between previous action and density was also significant (df=1, χ^2=57.46, p=3.45e-14). A full table of the regression results is available in the project's OSF directory.

We ran an additional, exploratory model that included a time-varying covariate for each participant's cumulative number of friends. We found that the effect of total friends on friendship requests was significant (df=1, $\chi^2 = 15.35$, p=9.41e-5), with higher friend counts associated with a reduced likelihood of sending a new request. This suggests that participant behavior aligns with the common sense assumption that we in some way "satiate" as we accumulate more friends, perhaps because we recognize that we can meet the need to nurture and maintain only so many friendships. However, even after accounting for existing total friends, note that the effect of social environments, namely that of friendliness (df=1, $\chi^2 = 99.31$, p < 2.2e-16) and density (df=1, $\chi^2 = 143.97$, p < 2.2e-16), remained significant.

3. Mixed-effects models for reaction times

A linear mixed model ANOVA showed the same effects of friendliness and density on reaction times as reported in the main text. The main effects of friendliness (df=1, χ^2=54.61, p=1.47e-13) and density (df=1, χ^2=59.41, 1.27e-14) were significant, and so was their interaction (df=1, χ^2=20.53, p=5.87e-06).

4. Happiness, balance, and liking slider ratings

At the end of each block, participants were asked three questions. The first question was "How much did you like this club?" and participants indicated their response on a sliding scale from "Not at all" to "Very Much". Slider position started at the midway point, and participants were required to move the slider at least once before their response could be submitted. The next question was "Did you find a good balance for sending friend requests in this club?" and their responses were taken on a continuous sliding scale from "No, not at all" to "Absolutely yes". The third and final question was "How do you feel after visiting this club?" and participants indicated their responses on a sliding scale from "Very unhappy" to "very happy". The purpose of these questions was to get a subjective assessment of how participants viewed the different environments and how they felt while in that club.

The average rating recorded in the online cohort (n=783) are shown in Supplementary Figure 1 below. A 2x2 ANOVA showed a significant effect of friendliness and density on self-reported happiness (friendliness: df=780, F=5485, p<2.2e-16, pes=0.88; density: df=780, F=17.77, p=2.78e-05, pes=0.022; interaction: df=780, F=20.66, p=6.37e-06, pes=0.026), balance (friendliness: df=781, F=3428.15, p<2.2e-12, pes=0.814; density: df=781, F=22.27, p=2.81e-06, pes=0.021; interaction: df=781, F=16.813, p=4.56e-05, pes=0.021), and liking (friendliness: df=782, F=6031.86, p<2.2e-12, pes=0.885; density: df=782, F=9.77, p=2.00e-03, pes=0.012; interaction: df=782, F=31.93, p=2.24e-08, pes=0.039). This shows these two fundamental features of the social environment affected participants in a way that they had awareness over and could report back – adding to the effects on request rates and RTs reported in the main part of the manuscript.

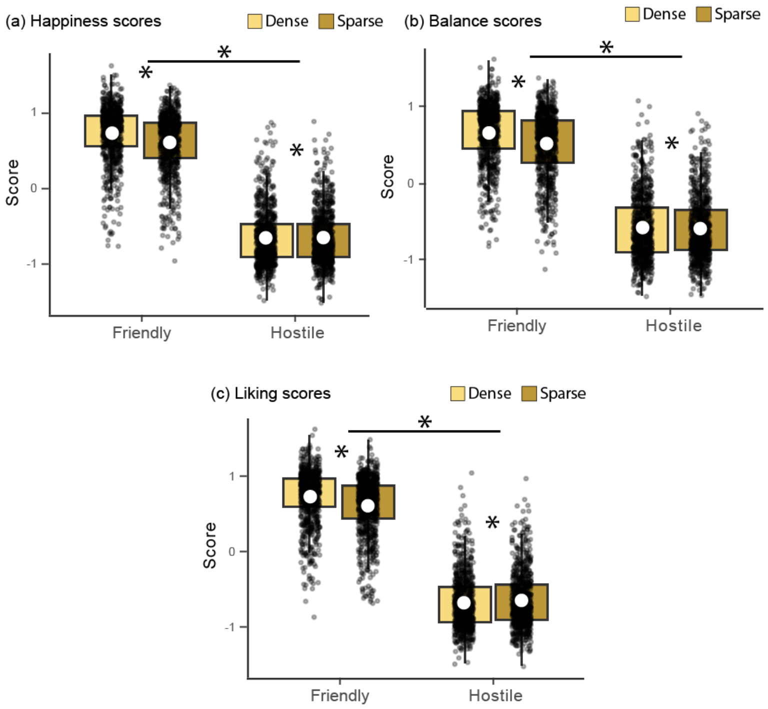

Figure 1. Subjective ratings (z-scored). (a) Happiness, (b) balance, and (c) liking ratings collected at the end of each block (white circle represents the mean, box boundaries indicate the interquartile range [IQR], encompassing the middle 50% of the data; whiskers extend to the furthest data points within 1.5 × IQR from the box boundaries).

5. Results of the factor analysis

After analysing an initial exploratory dataset (N=206), we pre-registered our hypothesis (https://osf.io/jf6vs/) that it would be possible, via factor analysis, to identify social and non-social factors and that some of these would be related to social connection seeking in the Friend Request Task. Six out of the seven pre-registered factors were observed in the confirmatory dataset: Social Thriving, Obsession/Compulsion, Impulsivity, Pleasure (reduced anhedonia), Sensation Seeking/Urgency, and Depression-Anxiety (see Fig.2 for an abbreviated list and supplementary Fig.2 for full factor loadings for all seven dimensions). We did not, however, identify a factor resembling the Social Pain Factor that we had found in the discovery data set analysis although, instead, we found some evidence for a factor that we termed social assurance.

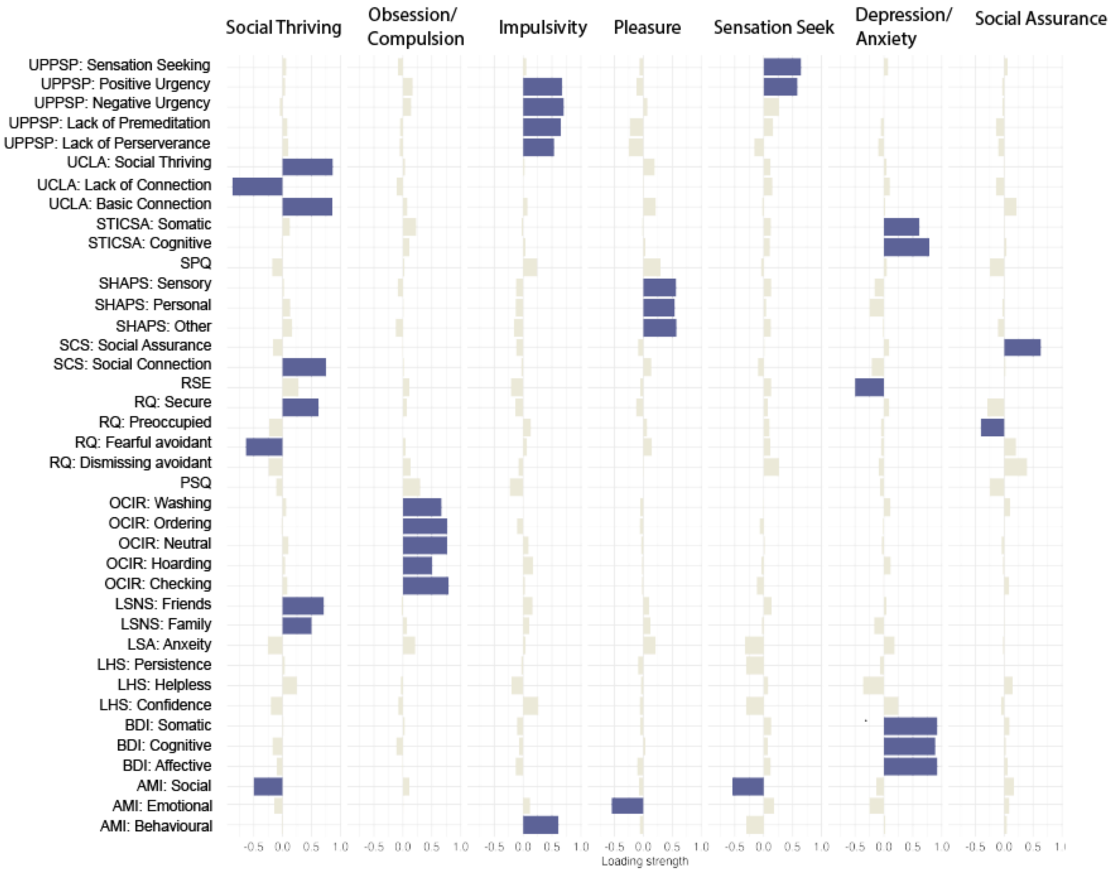

Figure 2. Factor structure and loadings. Results of a factor analysis revealed seven dimensions that captured aspects of participant's social and non-social personality and psychiatric profile. UPPSP: Urgency, Premeditation (lack of), Perseverance (lack of), Sensation Seeking, Positive Urgency, Impulsive Behaviour Scale. UCLA: UCLA loneliness scale. STICSA: State-Trait Inventory of Cognitive and Somatic Anxiety. SPQ: Social Pain Questionnaire. SHAPS: Snaith-Hamilton Pleasure Score. SCS: Social Connectedness Scale. RSE: Rosenberg Self-Esteem scale. RQ: Relationship Quotient. PSQ: Pain sensitivity questionnaire. OCIR: Obsessive Compulsive Inventory revised. LSNS: Lubben Social Network Scale. LSA: Liebowitz Social Anxiety Scale. LHS: Learned Helplessness Scale. BDI: Beck Depression Inventory. AMI: Apathy Motivation Index.

6. Pre-registered results on the relationship between task behaviour and mental health dimensions

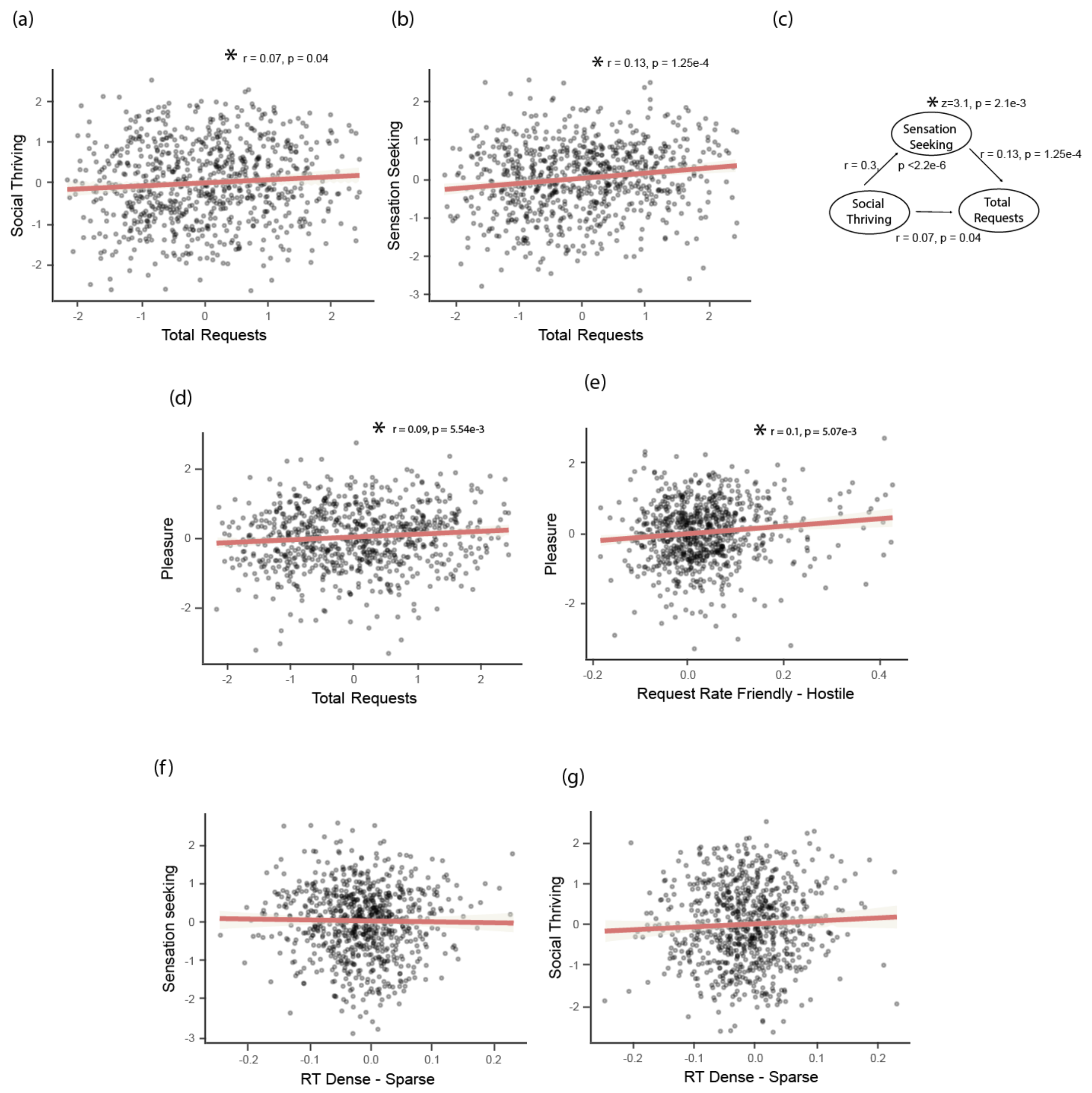

Figure 3. Relationship between social affiliation choices in the Friend Request Task and trans-diagnostic mental health dimensions. (a-b, d-g) Scatter plots show pre-registered relationships between Friend Request Task measures and transdiagnostic dimensions; (c) schematic showing the mediation relationship between social thriving, sensation seeking, and total friendship requests in the Friend Request Task.

In line with our pre-registered hypotheses derived from our discovery data set, we found that participants' social thriving scores were correlated with the total friend requests they submitted in the task (n=783, r=0.07, p=0.04; Fig. 3a). In other words, people who more frequently requested rather than skipped a friendship opportunity were those associated with higher social thriving scores. Participants' total requests were also related to their sensation seeking trans-diagnostic score (n=783, r=0.13, p=1.25e-4, Fig. 3b). We also found that the effect of total requests on social thriving was mediated by sensation seeking (n=783, z=3.1, p=2.1e-3, Fig. 3c). A pre-registered hypothesis that social pain was negatively related to total requests did not replicate, because, as noted, the factor itself did not replicate.

Next, the total number of requests made by participants was also related to their pleasure factor score (n=783, r=0.09, p=5.54e-3, Fig. 3d). Intriguingly, however, the pleasure factor was not just

related to the total number of requests made in the Friend Request Task but, in addition, it was related to the impact of a basic feature of social environment – the average friendliness – on social connection seeking (i.e., the request rate difference between friendly and hostile blocks: n=783, r=0.1, p=5.07e-3, Fig. 3e). In summary, of all the dimensions, the Pleasure (or reduced anhedonia) factor, therefore, may be particularly important in capturing how features of the background social environment influence whether people initiate social contact.

While the pre-registered predictions concerning relationships between mental health dimensions and Friend Request Task decision patterns were generally robust, as were RT patterns themselves, the relationships between mental health dimensions and RT patterns were not replicated (Fig. 3,f-g).

7. MRI behavioural effects that did not replicate

While our behavioural results were robust across two large online cohorts, not all results fully replicated in the smaller MR cohort which could be a power issue or related to the slightly slower task timings during the MR data acquisition. For full transparency, the equivalent results significant in the larger cohorts are shown here for the smaller sample.

In the online study, friendliness, density, and interactions had a significant effect on participant reaction time. This effect was not present in the MRI environment (2x2 ANOVA with two levels of friendliness and density; friendliness: df=25, F=0.004, p=0.953, pes=1.44e-4; density: df=25, F=0.462, p=0.503, pes=0.018, interaction: df=25, F=1.10, p=0.304, pes=0.042).

Similarly, in the online study, we found that previous trial outcome had a significant effect on subsequent choices. There was a trend for this effect in the MRI study (2x2x2 ANOVA with two levels of friendliness, density, and outcome; df=24, F=2.94, p=0.099, pes=0.109). In the online dataset, the previous trial outcome also interacted with friendliness of a block. This effect was not significant in the MRI dataset (df=24, F=1.69, p=0.206, pes=0.066).

In a similar vein, previous trial choices (request or skip) also had a significant effect on the request rates, which was not observed in the MRI dataset (2x2x2 ANOVA with 2 levels of friendliness, density, and previous action; df=25, F=0.881, p=0.357, pes=0.034). There was also an interaction between previous trial action and density in the online dataset, which was not observed in the MRI dataset (df=25, F=0.073, p=0.789, pes=0.003).

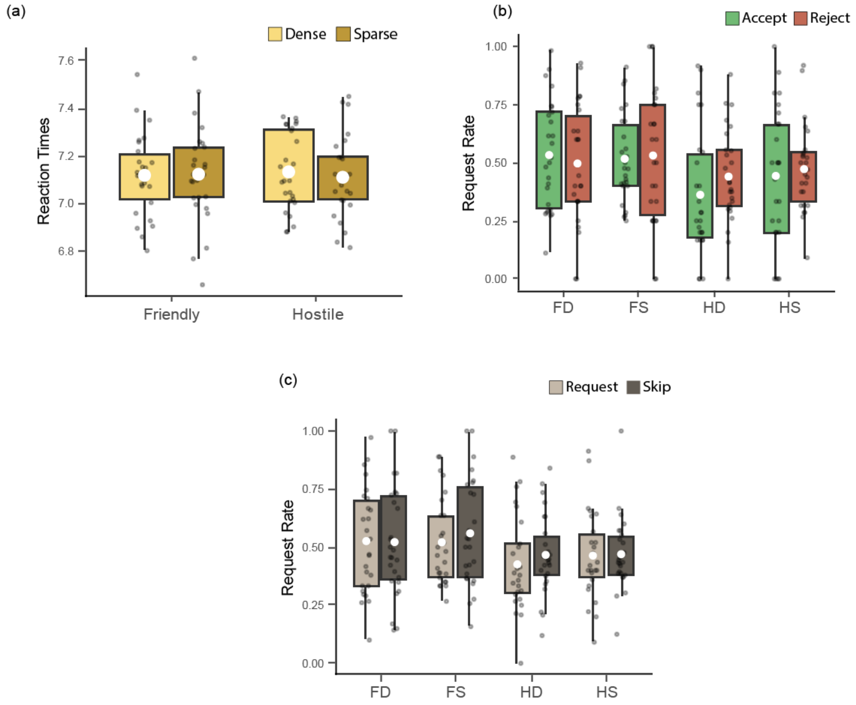

Figure 4: Behavioural effects in the smaller (n=24) MRI cohort for any effects that were significant in the larger online cohort, but not in the MRI study. (a) The effects of friendliness and density on RTs. Y-axis indicates average log-transformed RTs in the respective block type. (b) The effect of previous trial feedback on choice in the subsequent trial in different environment types. Colour indicates feedback received (accept/reject). (c) The effect of previous trial action on choice in the next trial. Colour indicates action (request/skip). Boxplots: white circle within each box represents the mean, while the box boundaries indicate the interquartile range [IQR], encompassing the middle 50% of the data. Whiskers extend to the furthest data points within 1.5 × IQR from the box boundaries.

Additional results related to the 7T-MRI cohort

8. DRN mask

The rationale for using a diffusion template was the finding that the DRN has lower fractional anisotropy (FA) values and the surrounding tissues have higher FA values, thus leading to an identifiable dark spot (approximate threshold: 3500 where the units are FA*10,000) in diffusion based FA images[38] (see supplementary Fig. 5; DRN mask also available at https://osf.io/jf6vs/).

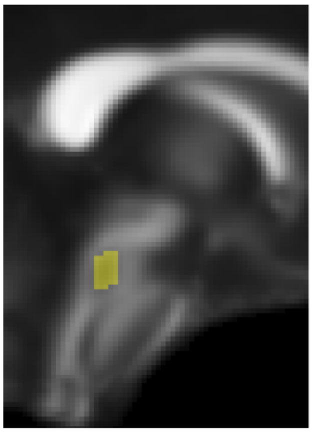

Figure 5: DRN mask overlayed over the standard FSL diffusion template.

9. Friendliness effects at outcome time

In the main part of the manuscript, the effects of the environment's friendliness were examined at the time of face presentation. Here, we additionally evaluated the effect of friendliness at the time of outcome, given reject/accept decisions may be processed differently as a function of the social environment. Using a mixed model ANOVA, with friendliness, density, region, and their interactions as fixed effects, and friendliness, density, and their interaction as random effects, we found a significant effect of friendliness (df=1, χ^2=9.86, p=1.68e-3), and a trend for density (df=1, χ^2=2.79, p=0.0947) on parameter estimates encoding outcome. There was also an interaction between friendliness and region (df=4, χ^2=27.55, p=1.538e-05). See supplementary Fig. 6 below.

Post-hoc tests were conducted to determine which regions were driving the friendliness effect. They revealed that a main effect of friendliness was present, after correcting for multiple comparisons, in the aI (df=1, χ^2=7.60, p=0.02) and area 9 (df=1, χ^2=9.36, p=0.01). Thus, we next examined whether the functional connectivity between area 9 and aI was related to model predicted pleasure scores in the HCP dataset (n=400) and found that there was a trend for such an association being present (r=0.1, p=0.05).

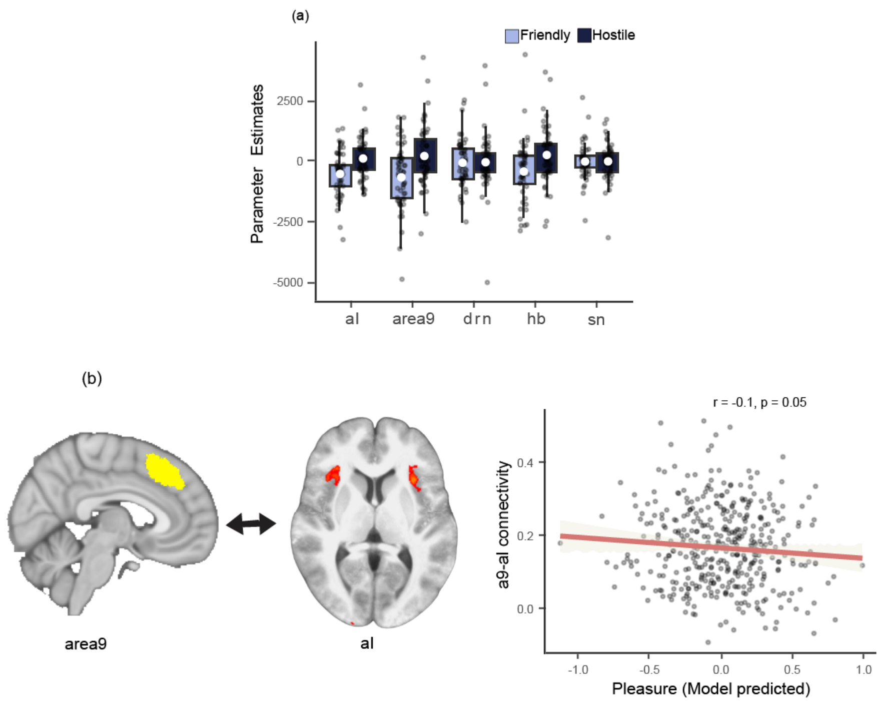

Figure 6: Friendliness effects evaluated at the time of outcome. (a) Boxplots indicate the effect of friendliness on parameter estimates encoding outcome. Colour indicates friendliness. Y-axis represents parameter estimates (white circle within each box represents the mean, while the box boundaries indicate the interquartile range [IQR], encompassing the middle 50% of the data. Whiskers extend to the furthest data points within 1.5 × IQR from the box boundaries). (b) Correlation plot showing model predicted pleasure scores on the x-axis and aI-area9 connectivity on the y-axis.

SN-aI resting-state connectivity relates to predicted pleasure factor score

So far, we have identified three main groups of findings. First, we found evidence that people's social connection seeking is influenced by basic statistical features of the social environment such as the density of social encounters and their average friendliness. Second, the same statistics of the social environment were correlated with activity patterns in several brain areas, notably in aI, DRN, and SN and interindividual variations in interactions between aI and the other two areas were associated with interindividual variation in the influence that the statistics of the social environment had on social affiliation seeking. Third, the influence of one statistic of the social environment – mean friendliness – on social affiliation seeking was linked to variation in the Pleasure (or reduced anhedonia) dimension from our factor analysis. In a final investigation we sought to test whether individual variation in this mental health dimension – Pleasure – was linked to variation in the same neural circuit.

To do this we reasoned that we would need access to a large MRI sample such as that provided by the Human Connectome Project (HCP)[39]. However, we also realized that it was unlikely that such a dataset would include assessment on the same questionnaires that were linked to the Pleasure scale. We therefore examined the questionnaire data that was available in the HCP

dataset and attempted to identify any relationship between the measures that were available and our Pleasure factor.

The HCP dataset contains scales measuring related concepts such as the NIH friendship and emotional support toolbox[36], the Ten item Personality Inventory (TIPI), comprising the Agreeableness, Openness to Experience, Emotional Stability, Extraversion, and Conscientiousness sub-scales[37]. We, therefore, collected scores on the same scales from the 783 participants who performed our behavioural experiments and assessed whether we could use machine learning to predict our factor-derived Pleasure scores from some combination of the NIH friendship and emotional support toolbox and TIPI scores. This was indeed the case (see supplementary Fig. 6), and we were able to create a quantitative translation of the questionnaire scores obtained from HCP participants into predictions of their scores on our Pleasure factor.

We made these behavioural Pleasure factor predictions for n=400 participants in the HCP data set. This subset of HCP n=400 participants was identified based on the availability of both questionnaire scores and cardiac/respiratory recordings that allowed physiological noise clean-up of the BOLD data to achieve more reliable brainstem BOLD signals (see [40,41] for more detail). We then examined whether the predicted Pleasure score could be related to variation in resting-state activity between either aI and SN or aI and DRN. We found that our machine learning model-derived Pleasure scores were significantly correlated with the functional connectivity between SN and aI (df=96, r=-0.11, t=-2.34, p=1.97e-2; Fig. 7). The greater the connectivity between SN and aI at rest, the lower the participant's Pleasure score.

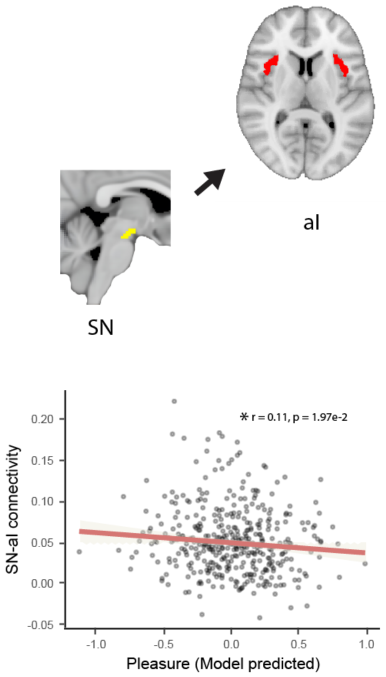

Figure 7. SN-aI resting-state connectivity relates to pleasure (or reduced anhedonia). Functional connectivity between the SN and aI inversely relates to model predicted Pleasure scores in a large group of participants from the Human Connectome Project (HCP; N=400). Greater connectivity between SN and aI was associated with lower pleasure and increased anhedonia.

Participants
For our resting-state analysis, a previously developed resting-state data set from a subset of n=400 HCP participants (males = 207, females = 193, mean age = 29) was used (for ethics, see

supplementary information). For full details on participant selection and additional preprocessing, see Klein-Flügge and colleagues[40,41].

Analysis

Participants from Experiment 1 were asked to complete the same questionnaires as HCP participants to establish a correspondence between questionnaire variables studied in Experiment 1 and those obtained in the HCP cohort. The process of model selection, fitting, and evaluation was performed using the 'tidymodels' package in R (see supplementary materials for details on model fitting). Having predicted Pleasure/reduced anhedonia for HCP participants based on their questionnaire data, we turned to their resting-state data. We used all four resting-state runs which had been acquired on a 3T Siemens scanner[42,43].

To extract the functional connectivity between SN and anterior insula for each individual, time series were extracted from the SN and the FOP4[44] region of the aI using the fully preprocessed concatenated dense rs-fMRI time series of each individual. The aI region used here (FOP4) was chosen as the best match of the insula parcellation provided by Glasser et al.[44] and the aI region found in our neuroimaging data. A functional connectivity score, indexed by the Pearson's r between the FOP4 and SN time course, was obtained for all participants to indicate the strength of resting-state connectivity between SN and aI. Hypothesised relationships were then evaluated for statistical significance using a simple Pearson's correlation test (Fig. 6). The significance threshold was set at 0.05.

10. Model evaluations for the pleasure factor

Four models (regularized linear regression, linear regression, neural networks, and random forests) were trained on a training dataset (n=587; obtained from the online Friend Request Task dataset) to predict the pleasure factor score. The winning model was selected that minimised the root mean squared error. The winning model was then evaluated on the testing dataset (n=196; remainder dataset which was not part of the training dataset); the model predicted pleasure factor score was significantly correlated to the true pleasure factor score (r=0.37, p=1.03e-07; see supplementary Fig. 8 below). This predictive model established here was then used to derive a Pleasure-like factor for the HCP participants in the last section of our main manuscript.

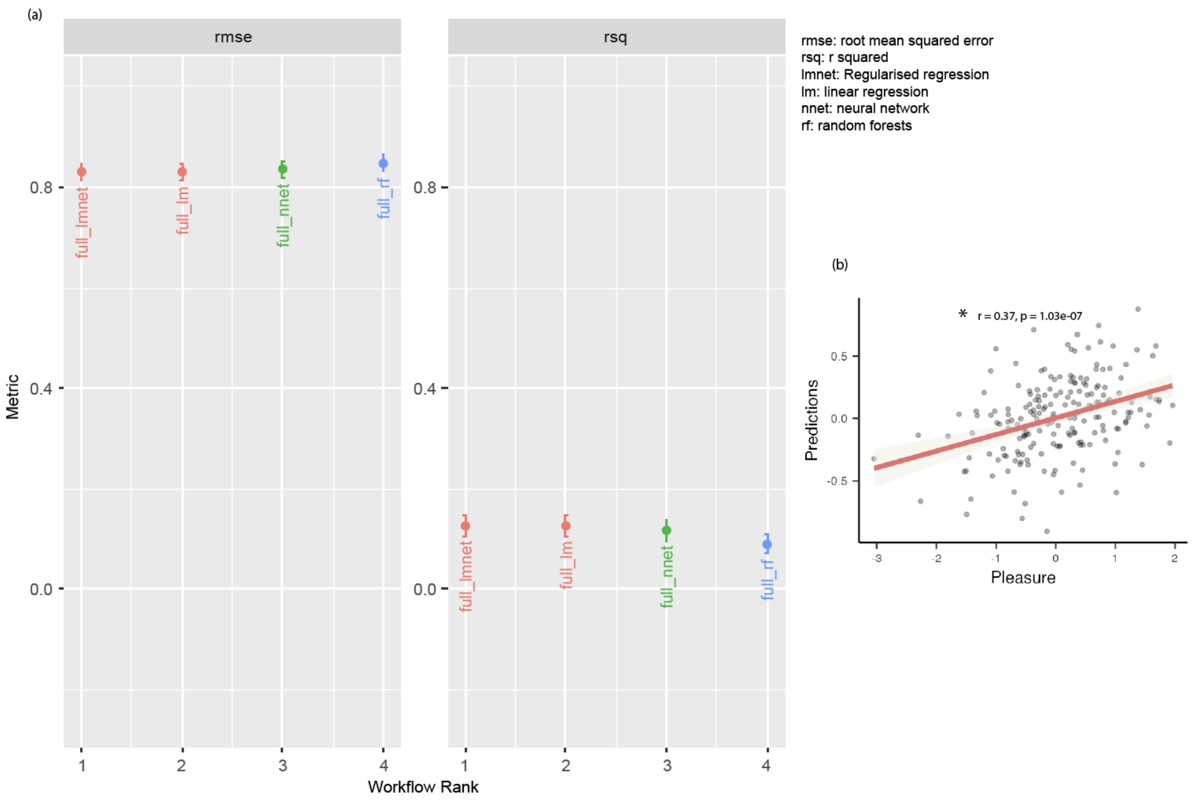

Figure 8: Model evaluations. (a) the left panel shows the root mean squared error, and the right panel shows the r squared for the four models used to predict the pleasure factor in the large online cohort (n=783). (b) correlation between the model predicted pleasure factor (y-axis) and true pleasure factor scores (x-axis), in the test dataset (n=196).

References

1. Faul, F., Erdfelder, E., Lang, A.-G. & Buchner, A. G*Power 3: A flexible statistical power analysis program for the social, behavioral, and biomedical sciences. *Behavior Research Methods* **39**, 175–191 (2007).
2. de Leeuw, J. R. jsPsych: A JavaScript library for creating behavioral experiments in a Web browser. *Behavior Research Methods* **47**, 1–12 (2015).
3. Lange, K., Kühn, S. & Filevich, E. "Just Another Tool for Online Studies" (JATOS): An Easy Solution for Setup and Management of Web Servers Supporting Online Studies. *PLoS ONE* **10**, e0130834 (2015).
4. Ma, D. S., Correll, J. & Wittenbrink, B. The Chicago face database: A free stimulus set of faces and norming data. *Behav Res* **47**, 1122–1135 (2015).
5. Lubben, J. E. Lubben Social Network Scale. American Psychological Association https://doi.org/10.1037/t10706-000 (2013).
6. Russel, D. UCLA Loneliness Scale (Version 3): Reliability, Validity, and Factor Structure. *Journal of Personality Assessment* **36**, ebi (1996).
7. Lee, R. M. & Robbins, S. B. Measuring Belongingness: The Social Connectedness and the Social Assurance Scales. *Journal of Counseling Psychology* **42**, 232–241 (1995).
8. Bartholomew, K. & Horowitz, L. M. Attachment Styles Among Young Adults: A Test of a Four-Category Model. *Journal of Personality and Social Psychology* **61**, 226–244 (1991).
9. Stangier, U., Schüller, J. & Brähler, E. Development and validation of a new instrument to measure social pain. *Sci Rep* **11**, 8283 (2021).

10. Liebowitz, M. R. Liebowitz-social-anxiety-scale - therapist administered.
11. Ang, Y.-S., Lockwood, P., Apps, M. A. J., Muhammed, K. & Husain, M. Distinct Subtypes of Apathy Revealed by the Apathy Motivation Index. *PLoS ONE* **12**, e0169938 (2017).
12. Beck, A. T., Steer, R. A., Ball, R. & Ranieri, W. F. Comparison of Beck Depression Inventories-IA and-II in Psychiatric Outpatients. *Journal of Personality Assessment* **67**, 588–597 (1996).
13. Snaith, R. P. *et al.* A scale for the assessment of hedonic tone. The Snaith-Hamilton Pleasure Scale. *British Journal of Psychiatry* **167**, 99–103 (1995).
14. Buckley, T. C., Parker, J. D. & Heggie, J. A psychometric evaluation of the BDI-II in treatment-seeking substance abusers. *J Subst Abuse Treat* **20**, 197–204 (2001).
15. Rosenberg, M. Society and the adolescent self-image. *Society and the Adolescent Self-Image* 1–326 (1965) doi:10.2307/2575639.
16. Quinless, F. & Nelson, M. Development of a measure of Learned Helplessness. *Nursing Research* **37**, (1988).
17. Foa, E. B. *et al.* The Obsessive-Compulsive Inventory: Development and validation of a short version. *Psychological Assessment* **14**, 485–496 (2002).
18. Cyders, M. A., Littlefield, A. K., Coffey, S. & Karyadi, K. A. Examination of a short English version of the UPPS-P Impulsive Behavior Scale. *Addictive behaviors* **39**, 1372–1376 (2014).
19. Grös, D. F., Antony, M. M., Simms, L. J. & McCabe, R. E. Psychometric properties of the State-Trait Inventory for Cognitive and Somatic Anxiety (STICSA): Comparison to the State-Trait Anxiety Inventory (STAI). *Psychological Assessment* vol. 19 369–381 (2007).
20. Ruscheweyh, R., Marziniak, M., Stumpenhorst, F., Reinholz, J. & Knecht, S. Pain sensitivity can be assessed by self-rating: Development and validation of the Pain Sensitivity Questionnaire. *Pain* **146**, 65–74 (2009).
21. Wittmann, M. K. *et al.* Global reward state affects learning and activity in raphe nucleus and anterior insula in monkeys. *Nat Commun* **11**, 3771 (2020).
22. Priestley, L. *et al. Dorsal Raphe Nucleus Controls Motivational State Transitions In Monkeys*. http://biorxiv.org/lookup/doi/10.1101/2024.02.13.580224 (2024) doi:10.1101/2024.02.13.580224.
23. Khalighinejad, N., Priestley, L., Jbabdi, S. & Rushworth, M. F. S. Human decisions about when to act originate within a basal forebrain-nigral circuit. *Proc Natl Acad Sci U S A* **117**, 11799–11810 (2020).
24. Trier, H. *et al.* Emotions and individual differences shape foraging under threat. Preprint at https://doi.org/10.31234/osf.io/v6u3y (2023).
25. Khalighinejad, N. *et al.* A Basal Forebrain-Cingulate Circuit in Macaques Decides It Is Time to Act. *Neuron* **105**, 370-384.e8 (2020).
26. Khalighinejad, N., Garrett, N., Priestley, L., Lockwood, P. & Rushworth, M. F. S. A habenula-insular circuit encodes the willingness to act. *Nat Commun* **12**, 6329 (2021).
27. Trier, H. A. *et al.* A distributed subcortical circuit linked to information-seeking about threat. *Proc Natl Acad Sci U S A* (in press).
28. Khalighinejad, N., Manohar, S., Husain, M. & Rushworth, M. F. S. Complementary roles of serotonergic and cholinergic systems in decisions about when to act. *Curr Biol* S0960-9822(22)00104-X (2022) doi:10.1016/j.cub.2022.01.042.
29. Priestley, L. *et al. Dorsal Raphe Nucleus Controls Motivational State Transitions in Monkeys*. http://biorxiv.org/lookup/doi/10.1101/2024.02.13.580224 (2024) doi:10.1101/2024.02.13.580224.
30. Neubert, F. X., Mars, R. B., Sallet, J. & Rushworth, M. F. Connectivity reveals relationship of brain areas for reward-guided learning and decision making in human and monkey frontal cortex. *Proc Natl Acad Sci U S A* (2015) doi:10.1073/pnas.1410767112.
31. Niv, Y., Daw, N. D., Joel, D. & Dayan, P. Tonic dopamine: opportunity costs and the control of response vigor. *Psychopharmacology* **191**, 507–520 (2007).

32. Anderson, J., Jenkinson, M. & Smith, S. Non-linear registration, aka spatial normalisation. FMRIB technical report TR07JA2. *FMRIB technical report TR07JA2* (2010).
33. Lindquist, M. A., Loh, J. M., Atlas, L. Y. & Wager, T. D. Modeling the Hemodynamic Response Function in fMRI: Efficiency, Bias and Mis-modeling. *Neuroimage* **45**, S187–S198 (2009).
34. Brooks, J. C. W. *et al.* Physiological noise modelling for spinal functional magnetic resonance imaging studies. *Neuroimage* **39**, 680–692 (2008).
35. Korponay, C., Janes, A. C. & Frederick, B. B. Brain-wide functional connectivity artifactually inflates throughout functional magnetic resonance imaging scans. *Nat Hum Behav* **8**, 1568–1580 (2024).
36. Cyranowski, J. M. *et al.* Assessing social support, companionship, and distress: National Institute of Health (NIH) Toolbox Adult Social Relationship Scales. *Health Psychology* **32**, 293–301 (2013).
37. Gosling, S. D., Rentfrow, P. J. & Swann, W. B. A very brief measure of the Big-Five personality domains. *Journal of Research in Personality* **37**, 504–528 (2003).
38. Bianciardi, M. *et al.* Toward an In Vivo Neuroimaging Template of Human Brainstem Nuclei of the Ascending Arousal, Autonomic, and Motor Systems. *Brain Connectivity* **5**, 597–607 (2015).
39. Smith, S. M. *et al.* Resting-state fMRI in the Human Connectome Project. *Neuroimage* **80**, 144–168 (2013).
40. Klein-Flügge, M. C. *et al.* Relationship between nuclei-specific amygdala connectivity and mental health dimensions in humans. *Nat Hum Behav* **6**, 1705–1722 (2022).
41. Jensen, D. E. A., Ebmeier, K. P., Suri, S., Rushworth, M. F. S. & Klein-Flügge, M. C. Nuclei-specific hypothalamus networks predict a dimensional marker of stress in humans. *Nat Commun* **15**, 2426 (2024).
42. Van Essen, D. C. *et al.* The WU-Minn Human Connectome Project: An overview. *NeuroImage* **80**, 62–79 (2013).
43. Smith, S. M. *et al.* Resting-state fMRI in the Human Connectome Project. *NeuroImage* **80**, 144–168 (2013).
44. Glasser, M. F. *et al.* A multi-modal parcellation of human cerebral cortex. *Nature* **536**, 171–178 (2016).
